# Assisted Fermentation by a Modified *Bacillus subtilis* Strain Producing Protease Improved the Quality of Sufu

**DOI:** 10.1002/fsn3.4673

**Published:** 2025-01-27

**Authors:** Junfei Xu, Aixiang Hou, Wenqi Li, Binbin Chen, Hong Wu, Huan Tan, Zhihong Xiao, Xianjin Wu, Juzuo Zhang

**Affiliations:** ^1^ College of Biological and Food Engineering/Key Laboratory of Research and Utilization of Ethnomedicinal Plant Resources of Hunan Province/Hunan Provincial Higher Education Key Laboratory of Intensive Processing Research on Mountain Ecological Food Huaihua University Huaihua China; ^2^ College of Food Science and Technology Hunan Agricultural University Changsha China; ^3^ State Key Laboratory of Utilization of Woody Oil Resources Hunan Academy of Forestry/Hunan Provincial Key Laboratory of Oils and Fats Molecular Structure and Function Changsha China; ^4^ Agricultural Products Processing Institute Hunan Academy of Agricultural Sciences/Hunan Food Testing and Analysis Center Changsha China

**Keywords:** *Bacillus subtilis*, flavor compound, nutrient, protease, sufu

## Abstract

Traditionally fermented sufu is popular because of its flavor, abundance of nutrients, and long shelf life. However, traditional sufu is difficult to produce via industrial processes because of dominant microorganism attenuation during fermentation. Herein, specific protease‐producing strains were isolated from traditional sufu. After strain identification, mutation, and domestication, the strains were applied in fermentation. The taste, texture, and nutrient and flavor components of the fermentation products were investigated via organoleptic, textural HPLC and HS‐GC‐IMS analyses. Results revealed that *Bacillus subtilis* (DF1) and the derived strains DF1v and DF1vd had increased protease activity relative to other strains. When these strains were applied for sufu fermentation, the production period significantly shortened to 6–8 days for pehtzes and to 20–26 days for postripening. The nutrient and flavor compound composition of both sufu pehtzes and products improved, including increases in water‐soluble proteins, amino acids, and substances with beany and umami aromas and decreases in nonbeneficial biogenic amines and moldy odor‐imparting substances. Among the strains, DF1vd showed the greatest benefits in sufu‐assisted fermentation. In summary, a modified *Bacillus subtilis* strain (DF1vd) producing protease was isolated, which improved the nutrient profile and flavor of sufu and shortened the production period.

## Graphical abstract

A dominant strain (DF1) producing proteases was isolated from traditional sufu. The specific strains derived from DF1 were obtained via UV mutagenesis (DF1v) and domestication under nutritional stress (DF1vd) conditions to facilitate the industrial use of the bacteria. These strains were further applied to assist in sufu fermentation. The nutrients and flavors of the prepared sufu products improved over those of naturally fermented sufu, and the fermentation period was shortened. These results can help guide efforts to improve the quality and stability of sufu during industrial production.

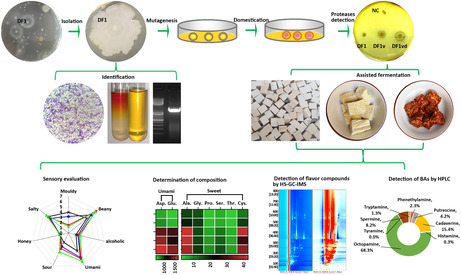

## 1 Introduction

Sufu, or *furu*, a traditional fermented soybean product originating from the Han Dynasty in China, is a popular flavor enhancer and appetizer because of its unique flavor, abundance of nutrients, and long shelf life (Han, Rombouts, and Nout 2001; Xu et al. 2020). Sufu can be prepared at home via the following processes. First, cleaned soybeans are soaked in water and ground into a slurry to separate the soybean protein and nutrients from other components, and the protein and nutrients are kept in a water‐soluble or colloidal form. The slurry is diluted, boiled, and filtered to prepare soymilk. The soymilk is coagulated and molded to produce tofu. The tofu is naturally fermented for approximately 20 days to produce the sufu pehtze, followed by flavor adjustment with different seasoners and additives and postripening for more than 1 month to produce the sufu product (Han, Rombouts, and Nout 2001). In the modern commodity market, there are slight differences in nutritional composition between commercial and homemade sufu because of differences in fermenting microorganisms and processing techniques (Li et al. 2021). Moreover, the production of toxins has always been a difficult issue and uncontrollable factor in natural fermentation, and comparatively artificial inoculation has some advantages in these aspects (Liu, Chen et al. 2022; Rohm et al. 2010). The mainstream approach involves enhancing the natural fermentation process through enrichment with microorganisms isolated from the environment, and microbial communities are the key factors affecting sufu quality. However, fermentation with pure cultures is limited because of the challenges associated with the long‐term storage of microorganisms applied in industrial sufu production (He and Chung 2020). Moreover, the safety and performance of fermentation microorganisms are unclear. Therefore, more attention should be given to the isolation and modification of fermentation microorganisms for sufu industrial production.

Before sufu production, the crude protein contained in tofu is weaved together to yield a certain gel strength (Liu, Sun, et al. 2022). Modification and hydrolysis of soybean proteins are important steps in sufu fermentation, which provide easily absorbed peptides, amino acids, and flavor‐inducing substances to promote human health (Li et al. 2021). These nutrients and flavor‐imparting compounds determine the market‐competitiveness of sufu products. In particular, the flavor and safety of sufu, which includes peptides, amino acids, and amines, are considered essential attributes that largely determine consumer acceptance of sufu (Xie et al. 2018). However, the production of these compounds depends on proteases from specific microorganisms, including fungi (e.g., *Mucor*, *Aspergillus*, and *Streptomyces*) and bacteria (e.g., *Bacillus* and *Lactobacillus*; Liang et al. 2021; Yao, Xu, Wu, Wang, Wang et al. 2021). Bacteria present several advantages in terms of the industrial production of sufu, for example, pure culture microorganisms offer controllability and operability during fermentation processes (Shahzad et al. 2020). However, the production of amines also raises many concerns, and compared to natural fermentation, control of initiation microorganisms in artificial inoculation could be used to avoid the accumulation of high concentrations of biogenic amines (Feng et al. 2016). In addition, it is difficult to find bacteria for the natural fermentation of sufu that can satisfy industrial application requirements due to the complexity of microbial metabolism and the generally low enzyme activity of microbes. Therefore, exploring the relationship between the composition of sufu and fermentation microorganisms is relevant.

To obtain safe, controllable, and operable bacterial resources for sufu industrial production, in the present study, specific bacteria that produce proteases were screened and isolated from traditional fermented sufu via casein plates, and the dominant strains were identified via physiological, biochemical, and molecular biological methods. To increase protease activity and facilitate industrial production, the obtained strains were subjected to ultraviolet light‐induced mutagenesis and nutritional stress to domesticate the strains. The modified strains were subsequently applied in assisted fermentation processes to improve the quality of the sufu product. The amino acids, biogenic amines, and volatile flavor compounds in the sufu were analyzed via an amino acid analyzer, high‐performance liquid chromatography (HPLC), and headspace gas chromatography ion mobility spectrometry (HS‐GC‐IMS). The results provide useful information for obtaining an in‐depth understanding of the unique flavor formation mechanism of sufu and serve as a reference for quality control in sufu fermentation processes.

## 2 Materials and Methods

### 2.1 Isolation of Specific Bacteria That Produce Proteases

Naturally fermented sufu was purchased from three different rural markets in Hunan Province, including the Huaihua Dongxing market, Shaoyang Gaosha market, and Xiangxi Qianzhou market. To preliminarily screen‐specific bacteria that produce proteases, approximately 25.00 g of sufu was added to a triangle flask that was loaded with 200 mL of casein enrichment medium (containing 1.0% casein, 0.3% beef extract, 0.3% glucose, 0.25% Na_2_HPO_4_, and 0.5% NaCl at pH 7.0) and cultured for 24 h at 37°C in a shaking 180 rpm incubator. This process was subsequently repeated three times by passaging with 20 mL of bacterial mixture. Then, 1 mL of the bacterial mixture was collected and added to 9 mL of normal saline to prepare 10 successive dilutions (10^−1^ ~ 10^−8^). Approximately 100 μL of the bacterial mixture from each diluted solution was collected and added to different culture plates to coat the surface of casein‐enriched solid media (including 2.0% casein, 0.3% beef extract, 0.25% Na_2_HPO_4_, 0.5% NaCl, and 1.5% agar at pH 7.0). The bacteria on these plates were cultured for 48 h at 37°C in a static incubator. Bacterial colonies with obvious transparent zones of protease activity were selected and preserved. A bacterial colony without a transparent zone was used as a negative control (NC). The specific bacteria were passaged on culture plates containing casein‐enriched solid media for further strain isolation and colony characterization.

### 2.2 Investigation of Bacterial Morphology

Bacterial morphology was investigated via the Gram staining method (Coico 2005). Briefly, bacterial cell samples removed from colonies via sterile tips were added and to 10 μL of normal saline and mixed in the center of a cleaned glass slide. After naturally drying, the sample on the glass slide surface was successively subjected to 5 min of fixation with absolute methanol, 2 min of dyeing with 2% ammonium oxalate/crystal violet solution, 1 min of dip‐dyeing with 1% potash iodide/iodine solution, 30 s of decolorizing with 95% ethanol, and 2 min of redyeing with 0.25% safranin solution. The sample on the glass slides was then gently washed with running water and spin‐dried. The dried glass slides were observed and photographed using an Olympus microscope.

### 2.3 Identification of Bacterial Biochemical and Biomolecular Characteristics

In accordance with the China National Standard Program and reference (Kumawat et al. 2021), the biochemical characteristics of the isolated dominant strains were determined via the Voges–Proskauer test (VP), methyl red test (MR), amylolysis test (Am), indole production test (IP), H_2_S production test (HS), gelatin hydrolysis test (GH), nitrate reduction test (NR), and catalase test (CT).

The genomic DNA of the dominant strains was extracted according to the manufacturer's instructions via a Rapid Bacterial Genomic DNA Isolation Kit (Cat. No. B518225, Sangon Biotech Co. Ltd., Shanghai, China). The 16S rDNA fragment was amplified according to the manufacturer's instructions via a 16S rDNA Bacterial Identification PCR Kit (Cat. No. B532063, Sangong). The sequencing and analysis of 16S rDNA were performed by Sangon Biotech Co. Ltd., via the universal 16S rDNA primers 7F (5’‐CAGAGTTTGATCCTGGCT‐3′) and 1540R (5’‐AGGAGGTGATCCAGCCGCA‐3′). Similar 16S rDNA sequences were analyzed and downloaded through an NCBI BLASTA search in the online database (http://www.ncbi.nlm.nih.gov/BLAST/). The downloaded sequences were subjected to complete alignment via ClustalX software with default parameters, and the phylogenetic tree was built via the maximum likelihood method on the basis of the Tamura–Nei model within MEGA11 software (Tamura, Stecher, and Kumar 2021).

### 2.4 Domestication of the Dominant Strain Producing Protease

The dominant strain with a high level of protease activity was modified through ultraviolet mutagenesis and nutritional stress domestication. Briefly, the dominant strain colony was selected and cultured in casein enrichment medium to the logarithmic growth phase. The bacterial cells were collected after 10 min of centrifugation at 3438 *g* and 4°C. Approximately 10^8^ bacterial cells in 2 mL of saline solution were added to a petri dish with a 9 cm diameter, and the dish was placed in a mutagenesis chamber at a distance of 20 cm from a 25 W ultraviolet light source for 120, 180, 240, or 300 s. The bacterial solutions were subjected to irradiation at 10‐fold dilutions of 10^−1^–10^−6^. One hundred microliters of bacterial solution from each dilution were added to different culture plates to coat the surface of a casein‐enriched solid medium. The bacteria in these plates were cultured for 48 h at 37°C in a stand incubator. The number of bacterial colonies on each plate surface was counted to determine the optimum ultraviolet irradiation time. The bacterial colony with the most transparent zones of protease activity was selected and preserved. The mutated strain was passaged more than 20 times in nutritional stress media (including 2.0% casein and 0.5% NaCl at pH 7.0).

### 2.5 Investigation of Bacterial Growth Status

One colony of bacteria, picked from the agar plate subjected to nutrient stress, was added to a shake flask containing fluid medium and cultured at 37°C in a 200 rpm shaker for 16 h. The OD_600nm_ of the cultured bacterial solution was measured from 0 to 16 at 2‐h intervals. Growth curves of the strains were generated with time on the *X*‐axis and OD_600nm_ on the *Y*‐axis.

### 2.6 Detection of Protease Activity

The specific strains were cultured in nutritional stress media for 24 h at 37°C in a shaking 180 rpm incubator. At 16 h of culture or at 2 h intervals from 0 ~ 22 h, an approximately 1.40 mL sample of bacterial solution was collected. To isolate the secreted protease, the liquid supernatant of the culture solution was collected through centrifugation at 3438 *g* and 4°C for 10 min. A total of 1.00 mL of crude enzyme was added to the measuring tube, followed by the addition of 1.00 mL of 2% casein solution. The measuring tube was incubated in a 40°C thermostat water bath for 10 min, and the enzymatic reaction was ended by the addition of 3.00 mL of 0.40 mol/L trichloroacetic solution to the test sample. A mixed solution consisting of 1.00 mL of normal saline, 3.00 mL of trichloroacetic solution, and 1.00 mL of casein solution was used as the control sample. The standard samples used to construct a calibration curve consisted of 20, 40, 60, 80, and 100 μg/mL tyrosine. After quiescence for 15 min at room temperature, 5.00 mL of 0.40 mol/L Na_2_CO_3_ solution and 1 mL of Folin's reagent were added to the measuring tube. The measuring tube was incubated in a 40°C thermostatic water bath for 10 min. Then, the optical density (OD) of the reaction solution was measured at 410 nm via an ultraviolet spectrophotometer (OD_410nm_).

To compare the protease activity of the mutant strain and domesticated strain with that of the initial strain, 2.5 μL of bacterial solutions with similar OD_600nm_ values were dropped at fixed locations onto the surface of casein‐enriched solid media. The bacteria in these plates were cultured for 48 h at 37°C in a static incubator. The external and internal diameters of the transparent zone of the strain were measured at 24 and 48 h, respectively. The external/internal diameter ratio of the transparent zone was calculated for the digitization of protease activity.

### 2.7 Application of Assisted Fermentation

The initial, mutant, and domesticated strains were cultured in liquid media at 37°C until the OD_600nm_ reached 1.20. The bacteria were collected after solution centrifugation, and 40 mL of distilled water was added to the mixture and stirred evenly to obtain a bacterial suspension. The tofu that was produced was lightly drained in a cool and dry environment, soaked in the bacterial suspension for 10 min, and drained for use. It was segmented and employed for testing in four groups: natural fermentation without further intervention as the natural fermentation control (Natural) group, initial strain‐assisted fermentation (DF1‐assisted) group, UV‐irradiated‐strain‐assisted fermentation (DF1v‐assisted) group, and domesticated strain‐assisted fermentation (DF1vd‐assisted) group. The tofu was subsequently incubated on a fermentation tray, covered with an insulated lid, and fermented at room temperature (approximately 22°C) for 6–10 days to obtain the sufu pehtzes. After a brief soak in cooking wine, the sufu pehtzes were mixed with salt and red pepper, canned and matured for 20 days to obtain the sufu products. The sufu pehtzes were collected from 0 days to 10 days of fermentation at 2 days intervals. Sufu products were collected after they were allowed to mature for 20 days.

### 2.8 Analysis of the Texture Profiles of the Sufu Pehtzes

The textural properties of the sufu pehtzes, including their hardness, cohesiveness, and springiness, were determined according to the methods of Czerner (Czerner et al. 2016) with a TA4/1000 probe using a texture analyzer (TMS‐PRO, Food Technology Corporation, USA). The rate at which the probe pressed downward was set to 1.5 mm/s before the measurement, the rate was maintained at 1.0 mm/s during the test, and the retraction rate was set to 1.5 mm/s, with a compression ratio of 30% and a trigger force of 0.5 N at 2‐s intervals.

### 2.9 Analysis of the Content of Water‐Soluble Protein, Amino Acid Nitrogen, Total Acid, and Hydrolyzable Amino Acid in the Sufu Pehtzes and Products

The contents of water‐soluble protein, amino acid nitrogen, total acid, and hydrolysis amino acids were determined according to the methods recommended by the Ministry of Agriculture of China (NY/T 1205–2006) and the Chinese National Standard (GB 5009.235–2016, GB 12456–2021). The formation of nonvolatile flavor compounds produced from hydrolyzable amino acids (HAAs) was investigated via an amino acid analyzer according to the Chinese National Standard GB 5009.124–2016. The samples of tofu and sufu were freeze‐dried into powders. A total of 2.000 g of sample was accurately weighed and brewed in a 250 mL conical bottle with 200 mL of boiling water, heated at 95°C for 10 min, mixed every 5 min, and then drained while hot. After the filtrate cooled to room temperature, 250 mL of water was added and mixed well, and an appropriate amount of sample solution was removed and filtered through a 0.45 μm water phase filter membrane for analysis. A sulfonic acid‐type cation exchange column was used with a mobile phase. The flow rate of the mobile phase was 0.35 mL/min, the sample volume was 20 μL, and the detection wavelengths were 570 nm and 440 nm.

### 2.10 Evaluation of the Sensory Quality of Sufu Products

The human sensory study was approved by the Science and Technology Ethical Committee of Huaihua University (2021EK04104). All the participants recruited from the student and faculty population were healthy and provided informed consent, and their rights and privacy were protected during the execution of the research. The aroma of the sufu products was blindly evaluated according to the requirements of the Institute of Food Science and Technology (IFST, UK) guidelines, ISO 8586‐2012, and the food industry standard (SB/T 10170–2007, China) by 20 trained evaluators (10 males and 10 females, aged 18–45). Each sample was evaluated three times at intervals of 30 s. A seven‐point number scale was used to evaluate the samples; a higher number meant a greater aroma intensity. The evaluators were ranked from 10 to 20 through stimulus training using seven types of aroma substances (mixture of sodium glutamate and inosine monophosphate for an umami smell, vinegar for a sour smell, vanillin water solution for a honey smell, low‐concentration soy sauce for a salty smell, fragments of fresh shiitake mushrooms for a moldy smell, soy sauce for a beany smell, and rice wine for an alcoholic smell). After the training, the evaluators scored the aroma intensity of the sufu products.

### 2.11 Analysis of Volatile Flavor Compounds in the Sufu Products

Volatile flavor compounds were investigated via an HS‐GC‐IMS system (Zeng et al. 2024; Shandong Haineng Scientific Instrument Co. Ltd.). A 1.00 g sample was incubated in a 20 mL headspace bottle at 60°C for 20 min, the oscillation rate was 500 r/min, and the injection needle temperature was 65°C. The final sample volume was 200 μL. N_2_ was used as the carrier gas (purity ≥ 99.9999%), and the nitrogen flow rate was maintained at 2 mL/min for 0–2 min; at 10 mL/min for 2–10 min; at 100 mL/min for 10–18 min; and at 150 mL/min for 18–30 min. Using n‐alkanes (C_4_ ~ C_9_) (a chromatographic reagent) as external standards, the retention indices (RIs) of the volatile compounds were determined, and the compounds were identified. Volatile compounds were determined via the RI and reference NIST library and IMS database. The relative content of a volatile compound was correlated with signal strength. Volatile flavor compounds were evaluated via the relative odor activity value (ROAV), and their contribution to overall flavor was determined. The calculation method was based on the following formula: ROAV_i_ = 100 (*C*_i_/*C*_max_) (*T*_max_/*T*_i_), where *C*_i_ and *T*_i_ are the relative concentrations and thresholds of a flavor component in water (Chen et al. 2023), respectively, which is the threshold reference. *T*_max_ and *C*_max_ are the thresholds and relative concentrations of volatile components with the highest odor activity value (OAV), respectively.

### 2.12 Analysis of Biogenic Amines in the Sufu Products

Biogenic amines (BAs) were analyzed via high‐performance liquid chromatography (HPLC) according to the Chinese National Standard GB 5009.208–2016. BA standards with purity ≥ 98%, which included tryptamine, β‐phenylethylamine, putrescine, cadaverine, histamine, tyramine, spermidine, octopamine, and spermine, were purchased from Shanghai Anpu Experimental Technology Co. Ltd. Dansulfonyl (chromatographically pure) was purchased from Shanghai Aladdin Biochemical Technology Co. Ltd. For the analysis, 5 g of sufu was added to a 50 mL centrifuge tube, 20 mL of 0.4 mol/L trichloroacetic acid (TCA) solution was added, and the sample was homogenized at high speed for 2 min. The sample was centrifuged, the entire liquid phase was transferred to a 100 mL plugged measuring cylinder, the precipitate was extracted again with 20 mL of 0.4 mol/L TCA solution, and the extracted liquid was added to the plugged measuring cylinder. Then, 4 mL of 4 mol/L sodium hydroxide solution and 4 mL of 0.5 mol/L sodium carbonate solution were added, the solution was adjusted to pH 11.0, 20 mL of trichloromethane and 20 mL of n‐butanol were successively added, and the mixture was shaken for 1 min and left undisturbed for 10 min, after which the upper water phase was poured out. Then, 1 mL of 1 mol/L hydrochloric acid was added to the lower organic phase, the mixture was evaporated by rotation to near‐dry conditions at 50°C, and 5 mL of 0.1 mol/L residue redissolved in hydrochloric acid was obtained. One milliliter of the above sample extract was added to a 5 mL plugged test tube, followed by the addition of 0.5 mL of 0.5 mol/L sodium carbonate solution and 0.5 mL of 5 g/L dansulfonyl chloracetone solution (0.5 g of dissolved dansulfonyl chloride in 100 mL of acetone). The solution was mixed well and heated in a 50°C water bath in the dark for 60 min. Then, 0.1 mL of concentrated ammonia water was added to remove excess derivatives, and the mixture was filtered through a 0.22 μm membrane and added to a chromatographic vial for HPLC analysis. The chromatographic conditions were as follows: sample size, 10 μL; flow rate, 1.0 mL/min; mobile phase A, 0.1% formic acid in water; mobile phase B, 0.1% formic acid in acetonitrile; program of 10% B at 0 min, 95% B at 10 min, 95% B at 30 min, 10% B at 30.1 min; column temperature, 35°C; and detection wavelength, 254 nm. Analyses were performed on a Shimadzu Shim‐pack Scepter C_18_ column (250 × 4.6 mm, 5 μm). All experiments were performed in triplicate, and the error was within 5%.

### 2.13 Statistical Analysis

Statistical analysis was performed via one‐way or two‐way ANOVA with post hoc multiple comparison (Bonferroni) after normal distribution tests were performed via the Statistical Package for Social Science (SPSS, Version 19.0; SPSS Inc., Chicago, IL, USA). The final data are shown as the mean ± standard deviation (SD), with statistical significance at *p* < 0.05 or *p* < 0.01.

## 3 Results

### 3.1 Four Strains Producing Proteases Were Isolated From Marketed Sufu

**FIGURE 1:**
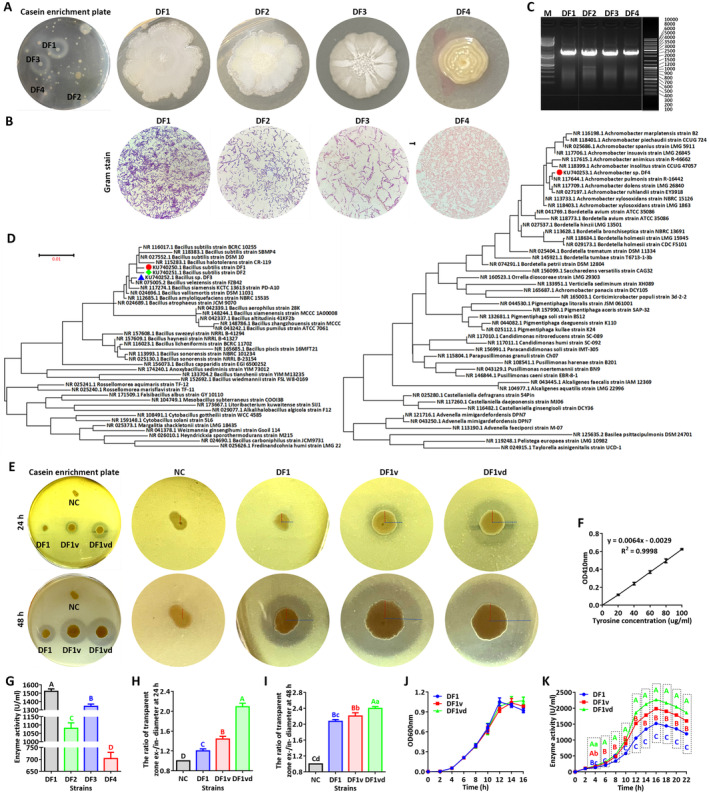
A specific strain (DF1) and derivative strains (DF1v and DF1vd) with the ability to produce proteases were isolated and identified from traditional sufu. (A) Primary screening plate and bacterial colonies of the DF1, DF2, DF3, and DF4 strains on secondary isolation plates. (B) The bacterial morphology of the DF1, DF2, DF3, and DF4 strains as determined by Gram staining. Scale bar: 10 μm. (C) PCR‐amplified fragments of 16S rDNA from the DF1, DF2, DF3, and DF4 strains. (D) Phylogenetic tree analysis of the 16S rDNA of the DF1, DF2, DF3, and DF4 strains. (E) Transparent zones corresponding to protease production in the DF1, DF1v, and DF1vd strains on casein‐enriched plates cultured for 24 and 48 h. The NC strain was used as a negative control for protease production. (F) The standard curve for protease analysis. (G) Protease activity of the DF1, DF2, DF3, and DF4 strains. (H–I) External/internal diameter ratio of the transparent zone corresponding to protease production in the DF1, DF1v, and DF1vd strains cultured for 24 and 48 h. The NC strain was used as a negative control. (J) Growth curves of the DF1, DF1v, and DF1vd strains after culture for 0 to 16 h were generated by determining the OD_600nm_. (K) Enzyme activity curves of the DF1, DF1v, and DF1vd strains during fermentation from 0 to 16 h. Different capital letters indicate highly significant differences (*p* < 0.01). Different lowercase letters indicate significant differences (*p* < 0.05).

After conditional screening and isolation, four pure strains were obtained from marketing sufu and named DF1, DF2, DF3, and DF4. The colony of the DF1 strain was in the form of an irregular and transparent circle with a dry and gray surface and a typical sideline around the colony. The colony of the DF2 strain was in the form of an irregular and transparent circle and had some yellow pigmentation, with a dry, white, and cottony surface. The colony of the DF3 strain was in the form of a regular and transparent circle with a white cottony surface. The colony of the DF4 strain was in the form of a regular and transparent circle and had 3–5 inner concentric circles with moist surfaces (Figure 1A). The fermented solution of the DF1 strain had the highest protease activity (1523.00 ± 28.79 U/mL) among the four strains, followed by that of the DF3 strain (1344.00 ± 26.80 U/mL), the DF2 strain (1081.00 ± 33.01 U/mL), and the DF4 strain (703.50 ± 26.01 U/mL), in that order (Figure 1A,G).

Biochemical analysis revealed that all four strains were negative to the VP and HS tests and positive to the MR and CT tests. Additionally, the DF1 strain was negative to the Am and IP tests and positive to the GH and CT tests. The DF2, DF3, and DF4 strains were positive to the Am and GH tests. Both the DF2 and DF3 strains were positive to the IP and NR tests. The DF4 strain was negative to the IP and NR tests (Table 1).

**TABLE 1:** Biochemical identification of specific strains that produce proteases.

Strains	VP	MR	Am	IP	HS	GH	NR	CT
DF1	−	+	−	−	−	−	+	+
DF2	−	+	+	+	−	+	+	+
DF3	−	+	+	+	−	+	+	+
DF4	−	+	+	−	−	+	−	+

Abbreviations: Am, amylolysis test; CT, catalase test; GH, gelatin hydrolysis test; HS, H_2_S production test; IP, indole production test; MR, methyl red test; NR, nitrate reduction test; VP, Voges‐Proskauer test.

Gram staining of the bacterial cells of DF1, DF2, and DF3 showed that all the bacteria stained blue and consisted of long‐chain bacilli, whereas the bacterial cells of the DF1 and DF2 strains had a deeper stain color than those of DF3, but the size and length of the bacterial cells of the DF1 and DF3 strains were larger and longer than those of the DF2 strain. In contrast, the bacterial cells of the DF4 strain stained red and consisted of coccobacilli (Figure 1B). Therefore, the DF1, DF2, and DF3 strains were gram‐positive bacteria, and the DF4 strain was a gram‐negative bacterium.

Four 16S rDNA fragments were obtained via DNA amplification and agarose electrophoresis (Figure 1C). The lengths of conserved sequences of the DF1, DF2, DF3, and DF4 strains were 1545, 1542, 1505, and 1493 bp, respectively. Through phylogenetic analysis, two completely different groups were constructed, with the DF1, DF2, and DF3 strains in one group and the DF4 strain in another group. The DF1, DF2, and DF3 strains were identified as *Bacillus subtilis* (ID: KU740250.1), *Bacillus subtilis* (ID: KU740251.1), and *Bacillus* sp. (ID: KU740252.1), respectively. The DF4 strain was identified as *Achromobacter* sp. (ID: KU740253.1) (Figure 1D). Among these strains, DF1, DF2, and DF3 are closely related, and all belong to *Bacillus*. The Bacillus, as a representative bacterial genus of probiotics, has the advantages of being non‐toxic to animals and producing safe food. DF4 belongs to *Achromobacter* and poses a safety hazard for dietary consumption. Because DF1 is a microorganism of the *Bacillus subtilis* and has a higher protease activity than DF2 and DF3, it was chosen as the starting strain for subsequent experiments.

### 3.2 The Protease Activity of the DF1 Strain Was Increased After Mutagenesis (DF1v) and Domestication (DF1vd)

The DF1 strain was subjected to UV‐induced mutagenesis, and the number of colony‐forming units (CFUs) per plate is shown in Table 2. At the same dilution, the CFU decreased with prolonged irradiation time. At the same intensity of UV irradiation, the CFU also decreased as the degree of dilution increased. When the irradiation time was 240 s, there were 22 viable bacterial clones, from which a mutant with a larger transparent circle than that of DF1 was obtained and passaged with stable inheritance. The mutant of the DF1 strain was named DF1v. DF1v was domesticated in nutritional stress media, and the resulting domesticated strain was named DF1vd.

**TABLE 2:** The colony‐forming units (CFUs) per plate after different UV irradiation duration in the gradient dilution of the DF1 strain with an initial OD600 nm of 1.037.

Dilution gradient	CFU per plate after different times of UV irradiation			
	120 s	180 s	240 s	300 s
10^−4^	Countless	Countless	410	168
10^−5^	134	89	22	5
10^−6^	18	3	0	0

The outer‐to‐inner diameter ratio of the transparent zone around the strain colonies significantly increased at both 24 and 48 h and were in the order DF1vd > DF1v > DF1 > NC (Figure 1E,H,I). There was no significant change in bacterial growth before the plateau phase. However, after the plateau phase, DF1v and DF1vd had better growth than DF1 did (Figure 1J). During fermentation, the protease activity in the DF1vd strain solution was significantly greater than that in the DF1 and DF1v strain solutions from 4 to 22 h. Additionally, the peak of protease activity in the DF1vd strain solution (2269.00 ± 31.59 U/mL) was significantly greater than that in the DF1v strain solution (1987.00 ± 29.75 U/mL) and DF1 strain solution (1523.00 ± 28.79 U/mL; Figure 1K).

### 3.3 Sufu Pehtzes–Assisted Fermentation Had More Stable Texture and Abundance of Crude Components at a Shorter Fermentation Period

**FIGURE 2:**
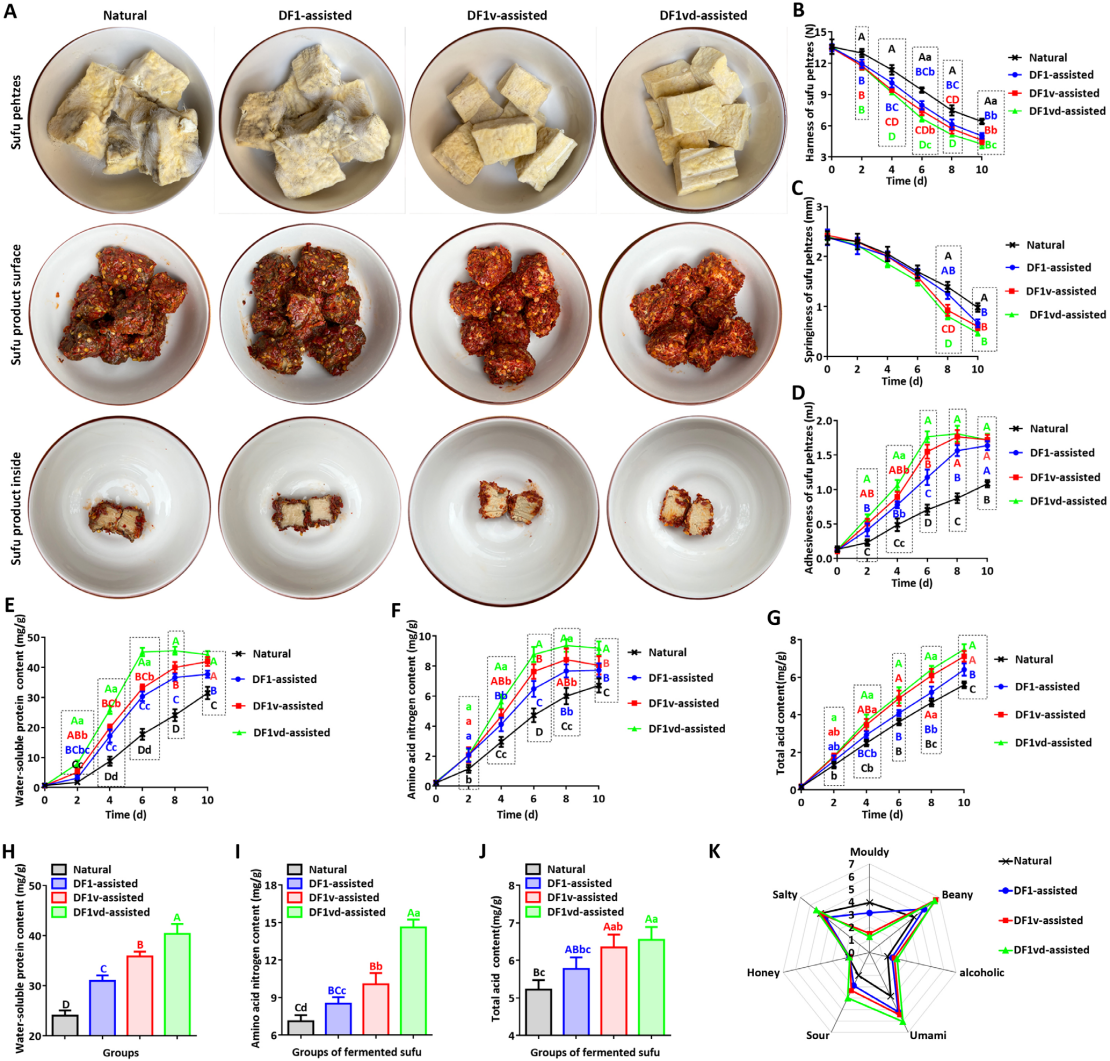
The sufu pehtze and sufu product crude component contents increased and their characteristics were enhanced through assisted fermentation. (A) The appearance of sufu pehtzes and the surface and interior of sufu products from the Natural, DF1‐assisted, DF1v‐assisted, and DF1vd‐assisted groups. (B) The trend in the hardness of sufu pehtzes from the Natural, DF1‐assisted, DF1v‐assisted, and DF1vd‐assisted fermentation processes. (C) The trend in the springiness of sufu pehtzes from the Natural, DF1‐assisted, DF1v‐assisted, and DF1vd‐assisted fermentation processes. (D) The trend in the cohesiveness of sufu pehtzes from the Natural, DF1‐assisted, DF1v‐assisted, and DF1vd‐assisted fermentation processes. (E) The trend in the water‐soluble content of sufu pehtzes from the Natural, DF1‐assisted, DF1v‐assisted, and DF1vd‐assisted fermentation processes. (F) The trend in amino acid nitrogen content of the sufu pehtzes from the Natural, DF1‐assisted, DF1v‐assisted, and DF1vd‐assisted fermentation processes. (G) The trend in the total acid content of sufu pehtzes from the Natural, DF1‐assisted, DF1v‐assisted, and DF1vd‐assisted fermentation processes. (H) Comparison of the water‐soluble content in the sufu products from the Natural, DF1‐assisted, DF1v‐assisted, and DF1vd‐assisted groups. (I) Comparison of amino acid nitrogen content in the sufu products from the Natural, DF1‐assisted, DF1v‐assisted, and DF1vd‐assisted methods. (J) Comparison of the total acid content in the sufu products from the Natural, DF1‐assisted, DF1v‐assisted, and DF1vd‐assisted groups. (K) Radar plot of the aroma characteristics of sufu produced in the Natural, DF1‐assisted, DF1v‐assisted, and DF1vd‐assisted groups. Different capital letters indicate highly significant differences (*p* < 0.01). Different lowercase letters indicate significant differences (*p* < 0.05). Natural fermentation: Natural fermentation; DF1‐assisted fermentation: DF1 strain‐assisted fermentation; DF1v‐assisted fermentation: DF1v strain‐assisted fermentation; DF1vd‐assisted fermentation: DF1vd strain‐assisted fermentation.

The structure and texture profiles of the sufu pehtzes fermented for 2, 4, 6, 8, and 10 days were analyzed. The pehtzes from all fermentation groups maintained the shape of the tofu used, but the pehtze surface in the DF1v‐assisted and DF1vd‐assisted groups had lower amounts of fungal hyphae than those in the Natural and DF1‐assisted groups (Figure 2A). The hardness of the sufu pehtzes obtained from all fermentation groups tended to decrease during the overall fermentation process. The sufu pehtzes in the Natural group were the hardest among the four groups, with hardness significantly different from those in the other groups at 2, 4, 6, 8, and 10 days (Natural > DF1‐assisted > DF1v‐assisted > DF1vd‐assisted). Compared with that of the Natural group, the hardness of the sufu pehtzes of the DF1‐assisted, DF1v‐assisted, and DF1vd‐assisted groups decreased more quickly, and the sufu pehtze of the DF1vd‐assisted group was the softest (Figure 2B). The springiness of sufu pehtzes from all fermentation groups also tended to decrease during the overall fermentation process. Moreover, compared with those in the Natural group, the springiness in the DF1‐assisted, DF1v‐assisted, and DF1vd‐assisted groups decreased more quickly, and the DF1v‐assisted group presented the greatest reduction. Among these fermentation methods, the springiness of sufu pehtzes significantly differed at 8 and 10 days (Natural > DF1‐assisted > DF1v‐assisted > DF1vd‐assisted), and the sufu pehtze was the most tender in the DF1vd group (Figure 2C). However, the cohesiveness of sufu pehtzes from all fermentation groups tended to increase throughout the fermentation process. Compared with those in the Natural group, the pehtze cohesiveness in the DF1‐assisted, DF1v‐assisted, and DF1vd‐assisted groups increased more quickly, and the DF1vd‐assisted group presented the greatest advantage in maintaining sufu shape (DF1vd‐assisted > DF1v‐assisted > DF1‐assisted>Natural), with significant differences at 2, 4, 6, 8, and 10 days (Figure 2D).

The crude components in the sufu pehtzes fermented for 2, 4, 6, 8, and 10 days were also analyzed. During fermentation, the water‐soluble protein content in the sufu pehtzes in all fermentation groups tended to increase. Compared with that in the sufu pehtzes from the Natural group, the water‐soluble protein content increased more quickly in the sufu pehtzes from the DF1‐assisted, DF1v‐assisted, and DF1vd‐assisted groups (DF1vd‐assisted > DF1v‐assisted > DF1‐assisted>Natural), showing significant differences at fermentation times of 2, 4, 6, 8 and 10 days. Meanwhile, the water‐soluble protein content reached a plateau after 6 days of fermentation in the sufu pehtzes from the DF1vd‐assisted group. This plateau was also observed after 10 days of fermentation in the sufu pehtzes of the DF1v‐assisted group. However, the water‐soluble protein content plateaued at a different value after 8 days of fermentation in the sufu pehtzes of the DF1‐assisted group, which had a value significantly lower than that of the plateau in the DF1vd‐assisted group (Figure 2E).

Moreover, the amino acid nitrogen content in the sufu pehtzes from all fermentation groups increased during the fermentation process. Compared with that in the sufu pehtzes from the Natural group, it increased more quickly in the sufu pehtzes from the DF1‐assisted, DF1v‐assisted, and DF1vd‐assisted groups (DF1vd‐assisted > DF1v‐assisted > DF1‐assisted>Natural), showing significant differences at fermentation times of 2, 4, 6, 8, and 10 days. However, it reached a plateau after 6 days of fermentation in the sufu pehtzes from the DF1vd‐assisted group. A plateau was also reached after 8 days of fermentation in the sufu pehtzes of the DF1‐assisted and DF1v‐assisted groups, but the value was significantly lower than that of the plateau of the DF1vd‐assisted group (Figure 2F).

The total acid content in the sufu pehtzes from all fermentation groups also increased during the fermentation process. There were similar trends in the total acid content of the sufu pehtzes of the DF1v‐assisted and DF1vd‐assisted groups, in which the levels were significantly greater than those in the sufu pehtzes of the DF1‐assisted and Natural groups at 4, 6, 8, and 10 days. The total acid content in the sufu pehtzes of the DF1‐assisted group was also significantly greater than that in the sufu pehtzes of the Natural group at fermentation times of 2, 4, 6, 8, and 10 days (Figure 2G).

### 3.4 Sufu Products–Assisted Fermentation Had Higher Content of the Crude Component and a Better Taste at a Shorter Postripening Period

After brief soak in cooking wine, the sufu pehtzes were mixed with salt and red pepper, canned, and matured for 20 days to produce sufu products. The salt fully penetrated the sufu products, and the red pepper was uniformly distributed over the sufu surface. The sufu products from the Natural and DF1‐assisted groups had dark red surfaces, while the surfaces of the sufu products from the DF1v‐assisted and DF1vd‐assisted groups had a brighter red color (DF1vd‐assisted > DF1v‐assisted > DF1‐assisted>Natural). When the sufu was cut into two parts along the centerline using a knife, it was found that the texture and surfaces of the sufu from the Natural and DF1‐assisted groups were relatively tight and smooth. However, the texture and surfaces of the sufu from the DF1v‐assisted and DF1vd‐assisted groups were relatively irregular, soft, tender, and cohesive (Figure 2A).

Compared with that in the sufu products from the Natural group, the water‐soluble protein content in the sufu products from the DF1‐assisted, DF1v‐assisted, and DF1vd‐assisted groups increased to different degrees, with significant differences (DF1vd‐assisted > DF1v‐assisted > DF1‐assisted>Natural; Figure 2H). The amino acid nitrogen content in the sufu products from the DF1‐assisted, DF1v‐assisted, and DF1vd‐assisted groups was also greater than that from the Natural group (DF1vd‐assisted > DF1v‐assisted > DF1‐assisted>Natural). There was no significant difference in amino acid nitrogen content between the DF1‐assisted and DF1v‐assisted groups (Figure 2I). The total acid content in the sufu products from the DF1v‐assisted and DF1vd‐assisted groups was greater than that from the Natural group. There was no significant difference in total acid content between the DF1‐assisted and DF1v‐assisted groups or between the Natural and DF1‐assisted groups. The total acid content in the sufu product from the DF1vd‐assisted group was significantly greater than that in the DF1‐assisted group (Figure 2J).

Seven characteristic smells of sufu were selected for sensory evaluation, including umami, sour, honey, salty, moldy, beany, and alcoholic. Compared with those in the Natural group, umami, sour and beany smells were enhanced in the sufu products from the DF1‐assisted, DF1v‐assisted, and DF1vd‐assisted groups, and those from the DF1vd‐assisted group presented the most obvious increases. The moldy smell in the sufu products from the DF1v‐assisted and DF1vd‐assisted groups was significantly weaker than that from the Natural and DF1‐assisted groups. However, there were no obvious differences in the honey, salty, or alcoholic smells of the sufu products among the different fermentation groups (Figure 2K).

### 3.5 Sufu Products–Assisted Fermentation Had a Higher Amount of Hydrolyzable Amino Acids (HAAs)

**FIGURE 3:**
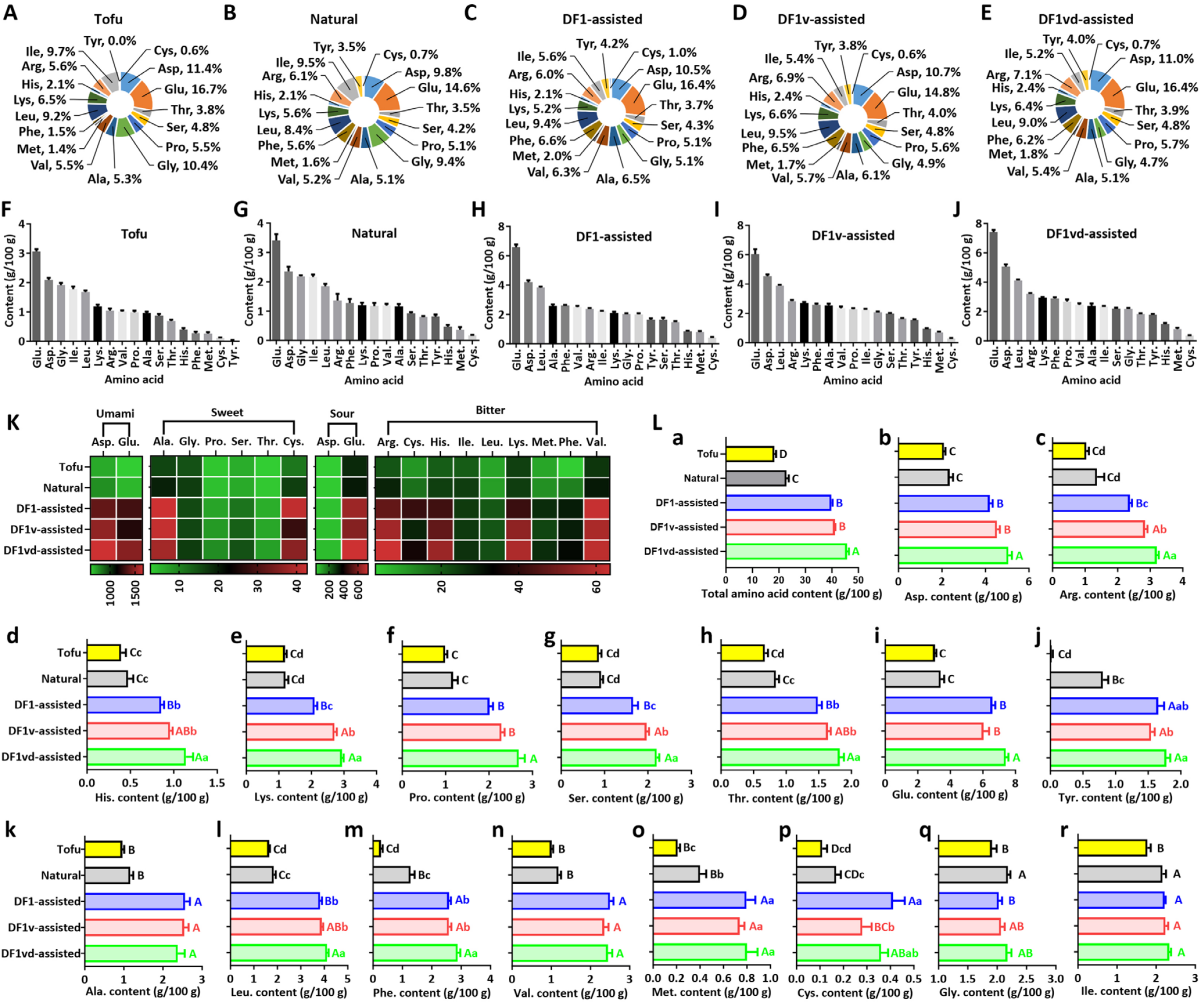
The proportion and content of hydrolyzable amino acids (HAAs) in the sufu products increased with assisted fermentation, which contributed to an improvement in product taste. (A) Proportion of HHAs in tofu. (B) Proportion of HHAs in the sufu products from the Natural fermentation group. (C) Proportion of HHAs in the sufu products from the DF1‐assisted group. (D) Proportion of HHAs in the sufu products from the DF1v‐assisted group. (E) Proportion of HHAs in the sufu products from the DF1vd‐assisted group. (F) The contents of HHAs in tofu. (G) The contents of HHAs in the sufu products from the Natural group. (H) The contents of HHAs in the sufu products from the DF1‐assisted group. (I) The contents of HHAs in the sufu products from the DF1v‐assisted group. (J) The contents of HHAs in the sufu products from the DF1vd‐assisted group. (K) Heatmap of the relationship between HHAs and taste, showing the following trend: Tofu<Natural < DF1‐assisted < DF1v‐assisted < DF1vd‐assisted for umami, sweet, sour, and bitter flavors, which is consistent with the color change. (L (a‐r)) The contents of 17 HAAs, namely, total amino acid (L(a)), aspartic acid (Asp.) (L(b)), arginine (Arg.) (L(c)), histidine (His.) (L(d)), lysine (Lys.) (L(e)), proline (Pro.) (L(f)), serine (Ser.) (L(g)), threonine (Thr.) (L(h)), glutamic acid (Glu.) (L(i)), tyrosine (Tyr.) (L(j)), alanine (Ala.) (L(k)), leucine (Leu.) (L(l)), phenylalanine (Phe.) (L(m)), valine (Val.) (L(n)), methionine (Met.) (L(o)), cysteine (Cys.) (L(p)), glycine (Gly.) (L(q)), isoleucine (Ile.) (L(r)), were significantly different among the tofu and sufu products from the Natural, DF1‐assisted, DF1v‐assisted, and DF1vd‐assisted groups. Different capital letters indicate highly significant differences (*p* < 0.01). Different lowercase letters indicate significant differences (*p* < 0.05). Natural fermentation: Natural fermentation; DF1‐assisted fermentation: DF1 strain‐assisted fermentation; DF1v‐assisted fermentation: DF1v strain‐assisted fermentation; DF1vd‐assisted fermentation: DF1vd strain‐assisted fermentation.

The HAA contents, including those of alanine (Ala.), arginine (Arg.), aspartic acid (Asp.), cysteine (Cys.), glutamic acid (Glu.), glycine (Gly.), histidine (His.), isoleucine (Ile.), leucine (Leu.), lysine (Lys.), methionine (Met.), phenylalanine (Phe.), proline (Pro.), serine (Ser.), threonine (Thr.), tyrosine (Tyr.), and valine (Val.), in tofu and in different sufu products were investigated. The HAA composition in the tofu or sufu from the Natural, DF1‐assisted, DF1v‐assisted, and DF1vd‐assisted groups is significantly different. For example, the proportion of Glu was 16.7%, 14.6%, 16.4%, 14.8%, and 16.4% of the total amino acids in tofu or sufu in the Natural, DF1‐assisted, DF1v‐assisted, and DF1vd‐assisted groups, respectively (Figure 3A–E). The HHA content in the tofu or sufu products from the Natural, DF1‐assisted, DF1v‐assisted, and DF1vd‐assisted groups is also significantly different. For example, the Glu contents were 3.06 ± 0.08 g/100 g, 3.41 ± 0.22 g/100 g, 6.58 ± 0.19 g/100 g, 6.04 ± 0.34 g/100 g, and 7.40 ± 0.17 g/100 g in tofu or sufu products from the Natural, DF1‐assisted, DF1v‐assisted, and DF1vd‐assisted groups, respectively (Figure 3F–J).

Taste activity values (TAVs) were used to evaluate the contribution of each volatile compound to the overall odor. The TAVs are calculated as the ratio between the measured concentration of taste compounds and their predetermined taste threshold values. The greater the TAVs are, the greater the contribution of volatile flavor compounds to the overall flavor of sufu. Among these amino acids, Asp. and Glu. contribute to an umami flavor. Several amino acids, including Ala., Gly., Pro., Ser., Thr., and Cys., contribute to a sweet flavor. Other amino acids, including Asp. and Glu., contribute to a sour flavor. The amino acids Arg., Cys., His., Ile., Leu., Lys., Met., Phe., and Val. contribute to a bitter flavor. During the natural fermentation and assisted fermentation processes, these flavors were significantly improved and showed the following trends: umami>sour > bitter>sweet; sufu>tofu; and DF1vd‐assisted > DF1v‐assisted > DF1‐assisted>Natural in the sufu products (Figure 3K). These results indicated that umami was the main flavor in the sufu products. The umami flavor in the sufu could counteract bitterness, thus providing a palatable flavor to the sufu.

Overall, the amount of HAAs in the sufu products significantly increased, with 45.71 ± 0.74 g/100 g in the DF1vd‐assisted group, followed by 41.15 ± 0.16 g/100 g in the DF1v‐assisted group, 39.84 ± 0.44 g/100 g in the DF1‐assisted group, 22.87 ± 0.85 g/100 g in the Natural group, and 18.32 ± 0.60 g/100 g in the tofu (Figure 3L(a)). Compared with those in tofu, the levels of HAAs in the sufu products were significantly greater (Figure 3L(a–r)). Compared with the sufu products from the Natural group, the contents of all the HAAs (except for Gly. (Figure 3L(q)) and Ile. (Figure 3L(r))) in the sufu products from the DF1‐assisted, DF1v‐assisted, and DF1vd‐assisted groups significantly increased (Figure 3L(b–r)). The content of HHAs, including Asp, Arg, His, Lys, Pro, Ser., and Thr. tended to increase in the order DF1‐assisted < DF1v‐assisted < DF1vd‐assisted groups (Figure 3L(b–h)). The Glu. content in the sufu products from the DF1vd‐assisted group was significantly greater than those from the DF1‐assisted and DF1v‐assisted groups (Figure 3L(i)). However, the contents of the HAAs Tyr, Ala, Leu, Phe, Val, Met, Cys, Gly., and Ile. were not significantly greater in the sufu products from the DF1vd‐assisted group than in those from the DF1‐assisted and DF1v‐assisted groups (Figure 3L(j–r)).

### 3.6 Sufu Products–Assisted Fermentation Had More Refinement Flavor With Improved Volatile Compound Composition

**FIGURE 4:**
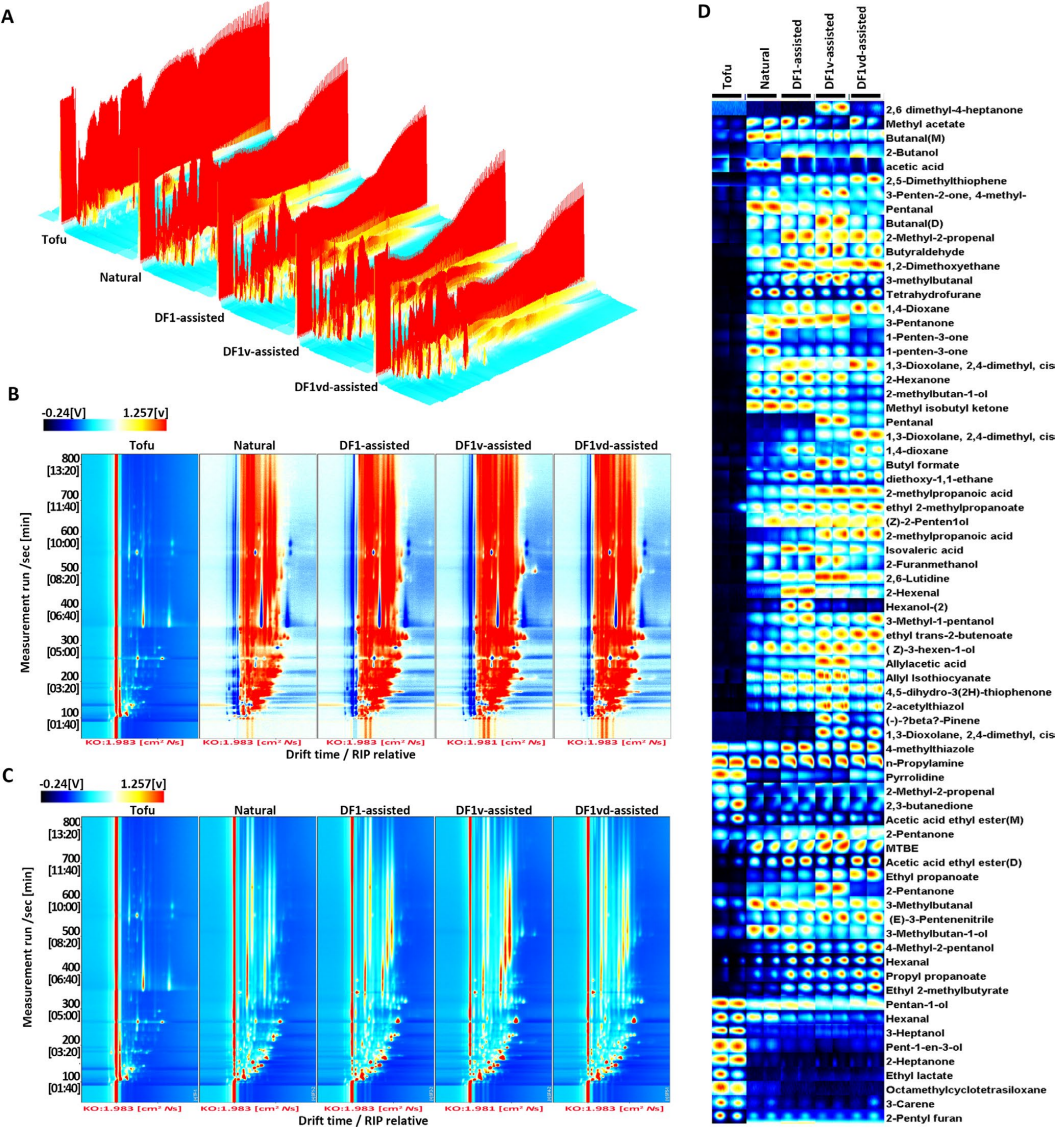
Flavor refinement occurred due to changes in volatile compounds after assisted fermentation. (A) 3D topographical images of volatile compounds obtained by HS‐GC‐IMS for tofu and different sufu products. (B) Comparison of volatile compounds in tofu and different sufu products via HS–GC–IMS (if the concentrations of volatile compounds were the same, the background after subtraction was white, while red indicated that the concentration of the substance was higher than the reference, and blue indicated that the concentration of the substance was lower than that of the reference). (C) Retention time (RT‐vertical axis), drift time (DT‐horizontal axis), and reaction ion peak (RIP‐vertical line). (D) Gallery plot of selected signal peak areas obtained from tofu and different sufu products via HS‐GC‐IMS. Each row represents the signal peaks selected in the sufu sample, and each column represents the signal peaks of the same volatile compound in different sufu products. An individual dot represents a volatile substance, and the color intensity represents the content of the volatile substance; the brighter the color is, the greater the content. Natural fermentation: Natural fermentation; DF1‐assisted fermentation: DF1 strain‐assisted fermentation; DF1v‐assisted fermentation: DF1v strain‐assisted fermentation; DF1vd‐assisted fermentation: DF1vd strain‐assisted fermentation.

The volatile flavor compounds (VOCs) in tofu and sufu products were determined via HS‐GC‐IMS. The results revealed that VOCs were more abundant in the sufu products than in tofu, and the VOCs present in the different sufu products were similar, as inferred from the color and peak intensity of the plots (Figure 4A). Nevertheless, some differences were found between the red points and the peak signal intensity. For ease of visualization, the top view was taken for detailed comparisons. A comparison of the different sufu products with tofu revealed that more red points were present in the different sufu products than in the tofu sample (Figure 4B). There were also differences among the sufu products from the Natural, DF1‐assisted, DF1v‐assisted, and DF1vd‐assisted groups, suggesting that VOCs are related to the composition of the fermenting microbial community (Figure 4C).

To highlight the differences in VOCs in the sufu products, the VOCs were analyzed three times in parallel, and their HS‐GC‐IMS fingerprint was obtained. As shown in the fingerprint plot (Figure 4D, Table 3), aldehydes, ketones, and alcohols were the major flavor compounds with low thresholds and strong odors, which contributed significantly to the overall flavor of sufu. A total of 39 volatile flavor compounds, including esters (11), alcohols (7), aldehydes (6), ketones (6), and furans (2), were identified from the tofu. A total of 66 volatile flavor compounds, including esters (11), alcohols (14), aldehydes (7), ketones (11), and furans (2), were identified from the sufu products.

**TABLE 3:** Contents of volatile flavor compounds in tofu and different sufu products detected by HS‐GC‐IMS.

No.	Compound	CAS	Formula	MW	RI	Rt [sec]	Dt [a.u.]	Threshold (mg/kg)	Tofu			Natural			DF1‐assisted			DF1v‐assisted			DF1vd‐assisted		
									Peak Intensity	%	ROAV	Peak Intensity	%	ROAV	Peak Intensity	%	ROAV	Peak Intensity	%	ROAV	Peak Intensity	%	ROAV
Alcohol																							
1	4‐Methyl‐2‐pentanol	C108112	C_6_H_14_O	102.20	742.30	227.09	1.56	2.50 ^a^	113.53 ± 1.62	0.25 ± 0.06	0.001	934.97 ± 102.85	0.61 ± 0.07	—	1788.92 ± 145.79	0.99 ± 0.08	0.01	653.74 ± 28.33	0.38 ± 0.02	—	529.61 ± 14.03	0.46 ± 0.01	—
2	3‐Methyl‐1‐butanol	C123513	C_5_H_12_O	88.10	731.40	218.80	1.24	0.30 ^b^	201.12 ± 35.74	0.44 ± 0.07	0.01	314.20 ± 22.59	0.21 ± 0.01	0.01	295.34 ± 23.58	0.16 ± 0.01	0.01	208.39 ± 16.28	0.12 ± 0.01	0.01	726.80 ± 40.17	0.63 ± 0.03	0.05
3	2‐Methyl‐1‐butanol	C137326	C_5_H_12_O	88.10	738.10	223.89	1.48	6.00 ^b^	—	—	—	808.97 ± 34.27	0.53 ± 0.02	—	1387.42 ± 58.38	0.76 ± 0.03	—	1311.24 ± 21.83	0.77 ± 0.01	—	870.62 ± 22.03	0.75 ± 0.02	—
4	(2Z)‐2‐Penten‐1‐ol	C1576950	C_5_H_10_O	86.10	764.20	244.70	1.44	0.72 ^a^	—	—	—	161.79 ± 8.88	0.11 ± 0.01	—	178.89 ± 6.07	0.10 ± 0.01	—	178.6 ± 18.39	0.10 ± 0.01	—	219.97 ± 45.46	0.19 ± 0.04	0.01
5	3‐Methyl‐1‐pentanol	C589355	C_6_H_14_O	102.20	848.70	326.74	1.60	0.01 ^a^	—	—	—	355.37 ± 61.84	0.23 ± 0.04	0.38	443.73 ± 11.89	0.24 ± 0.01	0.51	312.49 ± 9.77	0.18 ± 0.01	0.53	187.18 ± 68.13	0.16 ± 0.06	0.47
6	3‐Heptanol	C589822	C_7_H_16_O	116.20	889.50	376.04	1.33	0.24 ^a^	11213.20 ± 90.55	24.54 ± 0.55	0.99	2996.56 ± 171.72	1.97 ± 0.11	0.10	3365.97 ± 32.58	1.85 ± 0.02	0.12	2657.43 ± 158.77	1.56 ± 0.09	0.14	1503.88 ± 45.15	1.30 ± 0.04	0.12
7	1‐Penten‐3‐ol	C616251	C_5_H_10_O	86.10	676.10	181.39	0.94	0.35 ^b^	243.99 ± 22.39	0.53 ± 0.04	0.015	249.15 ± 16.76	0.16 ± 0.01	0.01	319.95 ± 87.23	0.18 ± 0.05	0.01	320.94 ± 142.34	0.19 ± 0.08	0.01	688.32 ± 14.22	0.59 ± 0.01	0.04
8	2‐Hexanol	C626937	C_6_H_14_O	102.20	826.20	302.51	1.57	4.00 ^b^	—	—	—	116.92 ± 19.84	0.08 ± 0.01	—	136.69 ± 23.82	0.08 ± 0.01	—	199.39 ± 18.29	0.12 ± 0.01	—	162.21 ± 5.34	0.14 ± 0.01	—
9	2‐Butanol	C78922	C_4_H_10_O	74.10	553.80	120.42	1.15	5.10 ^b^	—	—	—	207.81 ± 46.36	0.14 ± 0.03	—	293.05 ± 38.77	0.16 ± 0.02	—	169.05 ± 80.87	0.10 ± 0.05	—	342.71 ± 33.77	0.30 ± 0.03	—
10	2‐Methyl‐1‐propanol	C78831	C_4_H_10_O	74.12	577.40	130.26	1.35	8.00 ^b^	—	—	—	267.22 ± 1.57	0.18 ± 0.01	—	309.21 ± 26.69	0.17 ± 0.01	—	304.15 ± 44.20	0.18 ± 0.03	—	571.41 ± 50.14	0.49 ± 0.04	—
11	1‐Pentanol	C71410	C_5_H_12_O	88.10	764.70	245.11	1.25	5.00 ^b^	769.92 ± 25.62	1.69 ± 0.06	0.003	477.51 ± 29.87	0.31 ± 0.02	—	380.97 ± 44.94	0.21 ± 0.02	—	345.40 ± 64.35	0.20 ± 0.04	—	487.52 ± 42.16	0.42 ± 0.04	—
12	(E)‐3‐Hexen‐1‐ol	C928972	C_6_H_12_O	100.20	848.70	326.76	1.25	1.00 ^b^	2143.38 ± 30.83	4.69 ± 0.83	0.046	1615.84 ± 27.68	1.06 ± 0.02	0.01	1041.85 ± 155.53	0.57 ± 0.09	0.01	1167.61 ± 32.92	0.68 ± 0.02	0.02	1442.30 ± 166.87	1.24 ± 0.14	0.03
13	2‐Furan methanol	C98000	C_5_H_6_O_2_	98.10	834.80	311.61	1.37	1.00 ^b^	—	—	—	557.28 ± 13.51	0.37 ± 0.01	0.01	1531.18 ± 58.66	0.84 ± 0.03	0.01	965.70 ± 81.88	0.57 ± 0.05	0.01	277.99 ± 64.81	0.24 ± 0.06	0.01
14	Leaf alcohol	C928961	C_6_H_12_O	100.20	848.90	327.00	1.50	0.20 ^b^	—	—	—	736.01 ± 38.27	0.48 ± 0.03	0.03	1030.18 ± 74.62	0.57 ± 0.04	0.05	846.52 ± 10.15	0.50 ± 0.01	0.06	579.90 ± 12.17	0.50 ± 0.01	0.06
15	2‐Butoxyethanol	C111762	C_6_H_14_O_2_	118.20	904.40	395.81	1.21	2.60 ^b^	63.78 ± 12.94	0.14 ± 0.09	0.001		—	—	—	—	—	—	—	—	—	—	—
Aldehyde																							
16	1,1‐Diethoxyacetal	C105577	C_6_H_14_O_2_	118.20	713.50	205.88	1.12	0.10 ^b^	—	—	—	1378.45 ± 156.05	0.90 ± 0.10	0.11	2488.08 ± 146.17	1.37 ± 0.08	0.22	1622.27 ± 16.63	0.95 ± 0.01	0.21	1571.56 ± 182.60	1.35 ± 0.16	0.30
17	Pentanal	C110623	C_5_H_10_O	86.10	665.40	174.96	1.18	0.01 ^b^	—	—	—	400.93 ± 52.53	0.26 ± 0.03	0.40	284.46 ± 39.06	0.16 ± 0.02	0.32	800.96 ± 9.01	0.47 ± 0.01	1.30	548.21 ± 57.33	0.47 ± 0.05	1.30
18	Butanal	C123728	C_4_H_8_O	72.10	571.00	127.48	1.28	0.06 ^b^	—	—	—	2151.87 ± 137.85	1.41 ± 0.09	0.29	2348.84 ± 193.79	1.29 ± 0.11	0.34	2774.15 ± 37.67	1.62 ± 0.02	0.60	2622.28 ± 81.76	2.26 ± 0.07	0.83
19	2‐Hexenal	C505577	C_6_H_10_O	98.10	829.00	305.39	1.52	0.07 ^b^	—	—	—	163.55 ± 9.67	0.11 ± 0.01	0.02	148.98 ± 15.31	0.08 ± 0.01	0.02	190.09 ± 3.99	0.11 ± 0.01	0.04	135.38 ± 16.77	0.12 ± 0.01	0.04
20	3‐Methylbutyraldehyde	C590863	C_5_H_10_O	86.10	651.20	166.81	1.39	0.01 ^b^	265.10 ± 4.89	0.58 ± 0.01	0.704	3832.02 ± 232.57	2.51 ± 0.15	3.89	5225.41 ± 221.59	2.88 ± 0.12	5.68	5404.62 ± 456.50	3.16 ± 0.27	8.71	4287.38 ± 77.20	3.69 ± 0.07	10.17
21	Hexanal	C66251	C_6_H_12_O	100.20	788.50	265.86	1.26	0.02 ^b^	3037.08 ± 168.34	6.65 ± 0.01	3.228	9685.17 ± 109.95	6.36 ± 0.07	3.94	9814.57 ± 187.22	5.41 ± 0.10	4.27	8813.90 ± 300.64	5.16 ± 0.18	5.69	5272.30 ± 172.04	4.54 ± 0.15	5.01
22	2‐Methyl‐2‐propenal	C78853	C_4_H_6_O	70.10	568.10	126.29	1.22	0.01 × 10^−1b^	388.59 ± 52.08	0.85 ± 0.01	8.252	613.70 ± 29.75	0.40 ± 0.02	4.96	745.52 ± 35.41	0.41 ± 0.02	6.47	511.15 ± 52.15	0.30 ± 0.03	6.62	255.99 ± 32.77	0.22 ± 0.03	4.85
23	3‐Methyl‐2‐butenal	C107868	C_5_H_8_O	84.10	787.20	264.69	1.36	—	385.59 ± 50.80	0.84 ± 0.01	—	—	—	—	—	—	—	—	—	—	—	—	—
24	(E)‐2‐Pentenal	C1576870	C_5_H_8_O	84.10	743.60	228.10	1.10	0.10 ^b^	70.09 ± 14.21	0.15 ± 0.01	0.015	—	—	—	—	—	—	—	—	—	—	—	—
25	(E)‐Hept‐2‐enal	C18829555	C_7_H_12_O	112.20	967.50	492.18	1.27	0.01 × 10^−1b^	277.95 ± 3.89	0.61 ± 0.01	11.845	—	—	—	—	—	—	—	—	—	—	—	—
Ketone																							
26	2‐Heptanone	C110430	C_7_H_14_O	114.20	878.10	361.50	1.64	1.55 ^b^	2102.20 ± 83.16	4.60 ± 0.02	0.029	289.90 ± 61.98	0.19 ± 0.04	—	257.49 ± 26.77	0.14 ± 0.01	—	278.36 ± 30.65	0.16 ± 0.02	—	284.39 ± 14.09	0.25 ± 0.01	—
27	2,6‐Dimethyl‐4‐heptanone	C108838	C_9_H_18_O	142.20	974.70	504.61	1.78	8.00 ^b^	—	—	—	74.65 ± 15.71	0.05 ± 0.01	—	70.263 ± 3.80	0.04 ± 0.02	—	245.39 ± 30.90	0.14 ± 0.02	—	28.69 ± 2.56	0.02 ± 0.01	—
28	2‐Pentanone	C107879	C_5_H_10_O	86.10	674.20	180.22	1.12	0.30 ^b^	189.64 ± 20.52	0.42 ± 0.05	0.014	345.09 ± 21.87	0.23 ± 0.01	0.01	458.90 ± 54.61	0.25 ± 0.03	0.01	848.67 ± 147.54	0.50 ± 0.09	0.04	481.93 ± 91.84	0.42 ± 0.08	0.03
29	Tetrahydrothiophen‐3‐one	C1003049	C_4_H_6_OS	102.20	959.60	478.96	1.43	—	—	—	—	25234.15 ± 161.72	16.56 ± 0.11	—	30734.70 ± 322.66	16.93 ± 0.18	—	28867.55 ± 445.84	16.90 ± 0.26	—	15486.07 ± 97.54	13.34 ± 0.08	—
30	Methyl isobutenyl ketone	C141797	C_6_H_10_O	98.10	787.30	264.75	1.48	4.00 ^b^	—	—	—	646.96 ± 13.29	0.42 ± 0.01	—	614.08 ± 109.52	0.34 ± 0.06	—	897.42 ± 57.32	0.53 ± 0.03	—	934.89 ± 42.91	0.81 ± 0.04	—
31	4‐Methyl‐2‐pentanone	C108101	C_6_H_12_O	100.20	725.90	214.79	1.48	1.50 ^b^	—	—	—	423.49 ± 49.55	0.28 ± 0.03	—	447.93 ± 26.94	0.25 ± 0.01	—	479.45 ± 64.00	0.28 ± 0.04	—	601.64 ± 41.83	0.52 ± 0.04	0.01
32	1‐Penten‐3‐one	C1629589	C_5_H_8_O	84.10	687.40	188.49	1.31	0.12 × 10^−2b^	78.11 ± 23.05	0.17 ± 0.01	1.375	238.60 ± 9.06	0.16 ± 0.01	1.65	249.83 ± 108.34	0.14 ± 0.06	1.84	393.54 ± 53.41	0.23 ± 0.03	4.23	403.26 ± 98.69	0.35 ± 0.09	6.43
33	2,3‐Butanedione	C431038	C_4_H_6_O_2_	86.10	592.00	136.72	1.18	0.04 × 10^−1b^	1067.85 ± 8.89	2.34 ± 0.09	5.680	508.07 ± 12.68	0.33 ± 0.01	1.02	365.35 ± 91.11	0.20 ± 0.05	0.79	418.61 ± 15.09	0.25 ± 0.01	1.38	366.20 ± 43.67	0.32 ± 0.04	1.76
34	2‐Hexanone	C591786	C_6_H_12_O	100.20	744.10	228.46	1.52	0.25 ^b^	223.54 ± 5.59	0.49 ± 0.06	0.019	715.80 ± 66.47	0.47 ± 0.04	0.02	1282.39 ± 27.79	0.71 ± 0.02	0.04	908.85 ± 59.59	0.53 ± 0.03	0.05	443.67 ± 45.71	0.38 ± 0.04	0.03
35	3‐Pentanone	C96220	C_5_H_10_O	86.10	683.20	185.80	1.35	10.00 ^b^	—	—	—	145.01 ± 9.27	0.10 ± 0.01	—	441.45 ± 107.02	0.24 ± 0.06	—	403.26 ± 138.69	0.24 ± 0.08	—	363.21 ± 55.99	0.31 ± 0.05	—
36	2‐Butanone	C78933	C_4_H_8_O	72.10	578.00	130.51	1.25	3.00 ^b^	—	—	—	117.46 ± 12.69	0.08 ± 0.01	—	136.33 ± 4.28	0.08 ± 0.01	—	125.47 ± 18.62	0.07 ± 0.01	—	193.43 ± 25.77	0.17 ± 0.02	—
37	Hydroxyacetone	C116096	C_3_H_6_O_2_	74.10	678.90	183.12	1.23	0.20 ^b^	220.29 ± 12.29	0.48 ± 0.03	0.023	—	—	—	—	—	—	—	—	—	—	—	—
Ester																							
38	Propyl propanoate	C106365	C_6_H_12_O_2_	116.20	790.10	267.35	1.20	2.00 ^b^	693.97 ± 13.3	1.52 ± 0.01	0.007	2173.01 ± 39.80	1.43 ± 0.03	0.01	2252.15 ± 108.31	1.24 ± 0.06	0.01	1851.72 ± 87.86	1.08 ± 0.05	0.01	969.82 ± 82.09	0.84 ± 0.07	0.01
39	Ethyl propanoate	C105373	C_5_H_10_O_2_	102.10	694.30	192.93	1.45	0.04 × 10^−1b^	152.54 ± 39.07	0.33 ± 0.01	0.80	1528.52 ± 135.93	1.00 ± 0.09	3.10	1824.81 ± 77.13	1.01 ± 0.04	3.99	1084.43 ± 155.60	0.63 ± 0.09	3.47	280.43 ± 61.51	0.24 ± 0.05	1.32
40	Ethyl acetate	C141786	C_4_H_8_O_2_	88.10	613.80	147.11	1.10	0.10	6266.04 ± 86.4	13.71 ± 0.04	1.33	6581.60 ± 27.74	4.32 ± 0.02	0.54	7203.85 ± 464.31	3.97 ± 0.26	0.63	7547.35 ± 172.93	4.42 ± 0.10	0.98	6925.80 ± 214.94	5.97 ± 0.19	1.32
41	Butyl formate	C592847	C_5_H_10_O_2_	102.10	719.10	209.87	1.20	40.00 ^b^	—	—	—	288.78 ± 12.18	0.19 ± 0.01	—	175.49 ± 22.60	0.10 ± 0.01	—	433.91 ± 8.16	0.25 ± 0.01	—	274.63 ± 45.32	0.24 ± 0.04	—
42	Isobutyl formate	C542552	C_5_H_10_O_2_	102.10	659.20	171.32	1.51	30.00 ^b^	—	—	—	128.55 ± 9.88	0.08 ± 0.01	—	175.12 ± 49.18	0.10 ± 0.03	—	178.27 ± 87.30	0.10 ± 0.05	—	385.86 ± 1.77	0.33 ± 0.01	—
43	Allyl isothiocyanate	C57067	C_4_H_5_NS	99.20	897.40	386.41	1.38	0.05 ^a^	—	—	—	10978.33 ± 227.65	7.20 ± 0.15	1.94	17521.42 ± 104.74	9.65 ± 0.06	3.31	12178.05 ± 339.56	7.13 ± 0.20	3.42	4653.41 ± 112.08	4.01 ± 0.96	1.92
44	Ethyl trans‐2‐butenoate	C623701	C_6_H_10_O_2_	114.10	843.40	320.91	1.55	—	174.10 ± 48.19	0.38 ± 0.01	—	508.62 ± 13.61	0.33 ± 0.01	—	455.56 ± 4.28	0.25 ± 0.01	—	422.70 ± 17.98	0.25 ± 0.01	—	299.34 ± 117.25	0.26 ± 0.10	—
45	Methyl acetate	C79209	C_3_H_6_O_2_	74.10	537.70	114.17	1.18	550.00 ^b^	—	—	—	591.63 ± 146.19	0.39 ± 0.10	—	1606.83 ± 27.39	0.89 ± 0.02	—	359.28 ± 77.23	0.21 ± 0.05	—	2446.59 ± 52.35	2.11 ± 0.05	—
46	Ethyl isobutyrate	C7452791	C_7_H_14_O_2_	130.20	842.80	320.21	1.65	0.15 × 10^−3b^	605.49 ± 36.04	1.33 ± 0.01	86.08	1849.79 ± 31.25	1.21 ± 0.02	100.00	1716.11 ± 138.31	0.95 ± 0.08	100.00	1161.77 ± 67.73	0.68 ± 0.04	100.00	783.89 ± 5.98	0.68 ± 0.01	100.0
47	Ethyl lactate	C97643	C_5_H_10_O_3_	118.10	818.00	294.13	1.52	250.00	250.19 ± 18.05	0.55 ± 0.01	—	46.26 ± 4.90	0.03 ± 0.01	—	46.43 ± 5.06	0.03 ± 0.01	—	44.88 ± 3.95	0.03 ± 0.01	—	36.59 ± 6.70	0.03 ± 0.01	—
48	Ethyl 2‐methylpropanoate	C97621	C_6_H_12_O_2_	116.20	748.20	231.72	1.20	0.01 × 10^−2b^	468.82 ± 8.88	1.03 ± 0.01	100.00	593.31 ± 16.18	0.39 ± 0.01	48.35	779.03 ± 39.18	0.43 ± 0.02	67.89	491.15 ± 108.75	0.29 ± 0.06	63.97	262.00 ± 73.88	0.23 ± 0.06	50.74
49	Isopropyl methylphosphonofluoridate	C107448	C_4_H_10_FO_2_P	140.10	805.30	281.61	1.47	—	547.73 ± 30.99	1.20 ± 0.01	—	—	—	—	—	—	—	—	—	—	—	—	—
50	Ethyl hexanoate	C123660	C_8_H_16_O_2_	144.20	1016.90	584.07	1.34	0.01 ^b^	598.40 ± 71.45	1.31 ± 0.05	1.59	—	—	—	—	—	—	—	—	—	—	—	—
51	Leaf acetate	C3681718	C_8_H_14_O_2_	142.20	999.40	549.59	1.31	0.01 ^b^	319.10 ± 45.2	0.70 ± 0.01	0.56	—	—	—	—	—	—	—	—	—	—	—	—
52	Methyl pyromucate	C611132	C_6_H_6_O_3_	126.10	967.50	492.18	1.15	—	58.13 ± 19.96	0.13 ± 0.01	—	—	—	—	—	—	—	—	—	—	—	—	—
Furan																							
53	2‐Pentyl furan	C3777693	C_9_H_14_O	138.20	1003.40	557.28	1.25	0.48 × 10^−2b^	1630.39 ± 15.86	3.57 ± 0.09	7.22	852.00 ± 32.41	0.56 ± 0.02	1.45	854.25 ± 56.11	0.47 ± 0.03	1.55	723.80 ± 14.87	0.42 ± 0.01	1.93	1013.79 ± 26.98	0.87 ± 0.02	4.00
54	Tetrahydrofuran	C109999	C_4_H_8_O	72.10	630.50	155.55	1.23	18.00 ^a^	—	—	—	3538.92 ± 134.09	2.32 ± 0.09	—	4691.82 ± 69.10	2.58 ± 0.04	—	5632.68 ± 179.00	3.30 ± 0.10	—	8510.48 ± 395.11	7.33 ± 0.34	0.01
55	2‐Butylfuran	C4466244	C_8_H_12_O	124.20	887.90	373.99	1.18	0.01 ^a^	43.98 ± 14.58	0.10 ± 0.06	0.19		—	—		—	—		—	—	—	—	—
Others																							
56	Acetic acid	C64197	C_2_H_4_O_2_	60.10	611.20	145.81	1.15	50.00 ^b^	—	—	—	104.25 ± 1.17	0.07 ± 0.01	—	86.06 ± 19.06	0.05 ± 0.01	—	93.64 ± 44.31	0.05 ± 0.03	—	587.06 ± 55.21	0.51 ± 0.05	—
57	Allyl acetic acid	C591800	C_5_H_8_O_2_	100.10	885.90	371.40	1.44	—	—	—	—	418.60 ± 20.41	0.27 ± 0.01	—	516.07 ± 9.49	0.28 ± 0.01	—	649.57 ± 29.59	0.38 ± 0.02	—	285.91 ± 19.19	0.25 ± 0.02	—
58	3‐Methylbutanoic acid	C503742	C_5_H_10_O_2_	102.10	829.10	305.53	1.22	0.54 ^b^	—	—	—	493.06 ± 11.09	0.32 ± 0.01	0.01	305.32 ± 48.02	0.17 ± 0.03	—	403.59 ± 15.33	0.24 ± 0.01	0.04	626.37 ± 43.03	0.54 ± 0.04	0.02
59	2‐Methylpropionic acid	C79312	C_4_H_8_O_2_	88.10	771.10	250.48	1.17	3.00 ^b^	—	—	—	2107.18 ± 32.22	1.38 ± 0.02	0.01	1734.88 ± 26.22	0.96 ± 0.01	0.01	1903.89 ± 194.83	1.11 ± 0.11	0.01	1454.06 ± 111.17	1.25 ± 0.10	0.01
60	3‐Carene	C13466789	C_10_H_16_	136.20	1016.40	582.93	1.68	0.77 ^a^	2341.31 ± 136.60	5.12 ± 0.06	0.06	347.40 ± 132.53	0.23 ± 0.09	—	263.26 ± 56.90	0.15 ± 0.03	—	285.09 ± 26.44	0.17 ± 0.02	0.01	303.16 ± 48.02	0.26 ± 0.04	0.01
61	(−)‐β‐Pinene	C18172673	C_10_H_16_	136.20	976.70	508.19	1.63	—	326.66 ± 41.64	0.72 ± 0.06	—	688.95 ± 113.84	0.45 ± 0.07	—	841.00 ± 48.09	0.46 ± 0.03	—	1563.53 ± 109.63	0.92 ± 0.06	—	122.95 ± 27.13	0.11 ± 0.02	—
62	Styrene	C100425	C_8_H_8_	104.20	891.70	378.87	1.07	0.12 ^b^	—	—	—	773.09 ± 49.50	0.51 ± 0.03	0.05	610.44 ± 35.57	0.34 ± 0.02	0.04	453.09 ± 64.49	0.27 ± 0.04	0.05	240.98 ± 27.73	0.21 ± 0.02	0.04
63	2,4,6‐Trimethyl‐pyridine	C108758	C_8_H_11_N	121.20	1002.60	555.86	1.15	—	3303.83 ± 52.88	7.23 ± 0.09	—	6880.19 ± 30.16	4.51 ± 0.02	—	5248.30 ± 295.04	2.89 ± 0.16	—	5711.19 ± 12.16	3.34 ± 0.01	—	7552.14 ± 83.40	6.51 ± 0.07	—
64	2,6‐Dimethylpyridine	C108485	C_7_H_9_N	107.20	854.90	333.82	1.43	0.03 × 10^−1 a^	—	—	—	1044.33 ± 13.96	0.69 ± 0.01	2.85	1472.07 ± 53.89	0.81 ± 0.03	4.26	1401.15 ± 8.93	0.82 ± 0.01	6.03	761.51 ± 54.44	0.66 ± 0.05	4.85
65	2‐Acetylthiazol	C24295032	C_5_H_5_NOS	127.20	998.40	547.79	1.48	0.01 ^b^	—	—	—	35757.85 ± 153.61	23.46 ± 0.10	29.08	44521.00 ± 297.29	24.53 ± 0.16	38.73	41697.11 ± 634.77	24.41 ± 0.37	53.85	14151.88 ± 71.99	12.19 ± 0.06	26.89
66	4‐Methylthiazole	C693958	C_4_H_5_NS	99.20	814.90	291.02	1.04	0.02 ^b^	—	—	—	2329.08 ± 9.68	1.53 ± 0.01	0.95	2093.89 ± 58.76	1.15 ± 0.03	0.91	1870.05 ± 74.08	1.09 ± 0.04	1.20	1122.16 ± 18.84	0.97 ± 0.02	1.07
67	Octamethylcyclotetrasiloxane	C556672	C_8_H_24_O_4_Si_4_	296.60	1002.20	555.00	1.68	—	152.78 ± 21.07	0.33 ± 0.01	—	31.45 ± 4.34	0.02 ± 0.01	—	30.66 ± 1.30	0.02 ± 0.01	—	28.10 ± 0.57	0.02 ± 0.01	—	35.17 ± 4.30	0.03 ± 0.01	—
68	Methyl tert‐butyl ether	C1634044	C_5_H_12_O	88.10	533.20	112.47	1.13	0.15 × 10^−1b^	3663.65 ± 71.61	8.02 ± 0.06	5.19	5799.49 ± 216.12	3.81 ± 0.14	3.15	5784.35 ± 361.14	3.19 ± 0.20	3.36	7090.12 ± 282.76	4.15 ± 0.17	6.10	7651.68 ± 52.81	6.59 ± 0.05	9.69
69	1,4‐Dioxan	C123911	C_4_H_8_O_2_	88.10	661.40	172.61	1.33	2.90 ^a^	—	—	—	1388.93 ± 48.40	0.91 ± 0.03	—	2981.62 ± 288.06	1.64 ± 0.16	0.01	4665.25 ± 153.97	2.73 ± 0.09	0.02	6510.88 ± 51.27	5.61 ± 0.04	0.04
70	(E)‐3‐Pentenenitrile	C16529661	C_5_H_7_N	81.10	696.90	194.63	1.15	—	610.26 ± 33.68	1.34 ± 0.07	—	652.44 ± 44.15	0.43 ± 0.03	—	737.49 ± 33.97	0.41 ± 0.02	—	446.57 ± 249.67	0.26 ± 0.15	—	218.52 ± 35.79	0.19 ± 0.03	—
71	1,4‐Xylene	C106423	C_8_H_10_	106.20	865.10	345.69	1.06	8.70 ^b^	—	—	—	1967.04 ± 168.35	1.29 ± 0.11	—	1686.24 ± 79.19	0.93 ± 0.04	—	1354.93 ± 31.10	0.79 ± 0.02	—	306.58 ± 42.41	0.26 ± 0.04	—
72	2,5‐Dimethylthiophene	C638028	C_6_H_8_S	112.20	856.80	335.98	1.07	3.00 ^b^	—	—	—	421.17 ± 40.10	0.28 ± 0.03	—	336.46 ± 25.06	0.19 ± 0.01	—	248.17 ± 42.49	0.15 ± 0.02	—	61.99 ± 11.06	0.05 ± 0.01	—
73	2,4‐Dimethyl‐1,3‐Dioxolane cis	C3390123	C_5_H_10_O_2_	102.10	697.40	194.97	1.39	—	—	—	—	1007.24 ± 56.06	0.66 ± 0.04	—	475.26 ± 84.01	0.26 ± 0.05	—	1822.01 ± 65.85	1.07 ± 0.04	—	782.20 ± 8.37	0.67 ± 0.01	—
74	Pyrrolidine	C123751	C_4_H_9_N	71.10	686.40	187.82	1.05	20.20 ^a^	—	—	—	311.11 ± 20.70	0.20 ± 0.01	—	239.85 ± 99.47	0.13 ± 0.05	—	155.97 ± 8.40	0.09 ± 0.01	—	145.97 ± 23.96	0.13 ± 0.02	—
75	Propylamine	C107108	C_3_H_9_N	59.10	542.20	115.87	1.23	0.18 ^b^	—	—	—	3186.20 ± 80.51	2.09 ± 0.05	0.14	3321.38 ± 144.36	1.83 ± 0.08	0.16	2960.20 ± 37.81	1.73 ± 0.02	0.21	3262.61 ± 39.93	2.81 ± 0.03	0.34
76	1,2‐Dimethoxyethane	C110714	C_4_H_10_O_2_	90.10	621.00	150.69	1.29	—	—	—	—	257.40 ± 13.97	0.17 ± 0.01	—	325.83 ± 45.00	0.18 ± 0.02	—	294.94 ± 129.20	0.17 ± 0.08	—	180.05 ± 59.48	0.16 ± 0.05	—
77	Camphene	C79925	C_10_H_16_	136.20	946.80	458.27	1.20	30.00 ^a^	423.85 ± 14.92	0.93 ± 0.09	—		—	—		—	—		—	—	—	—	—

*Note:* The a indicates the threshold researched from https://www.vcfonline.nl/VcfHome.cfm. The b indicates the threshold researched from “Compilation of Odor Thresholds for Compounds”.

Abbreviations: MW, molecular weight; RI, retention index; Rt, retention time, sec.

**FIGURE 5:**
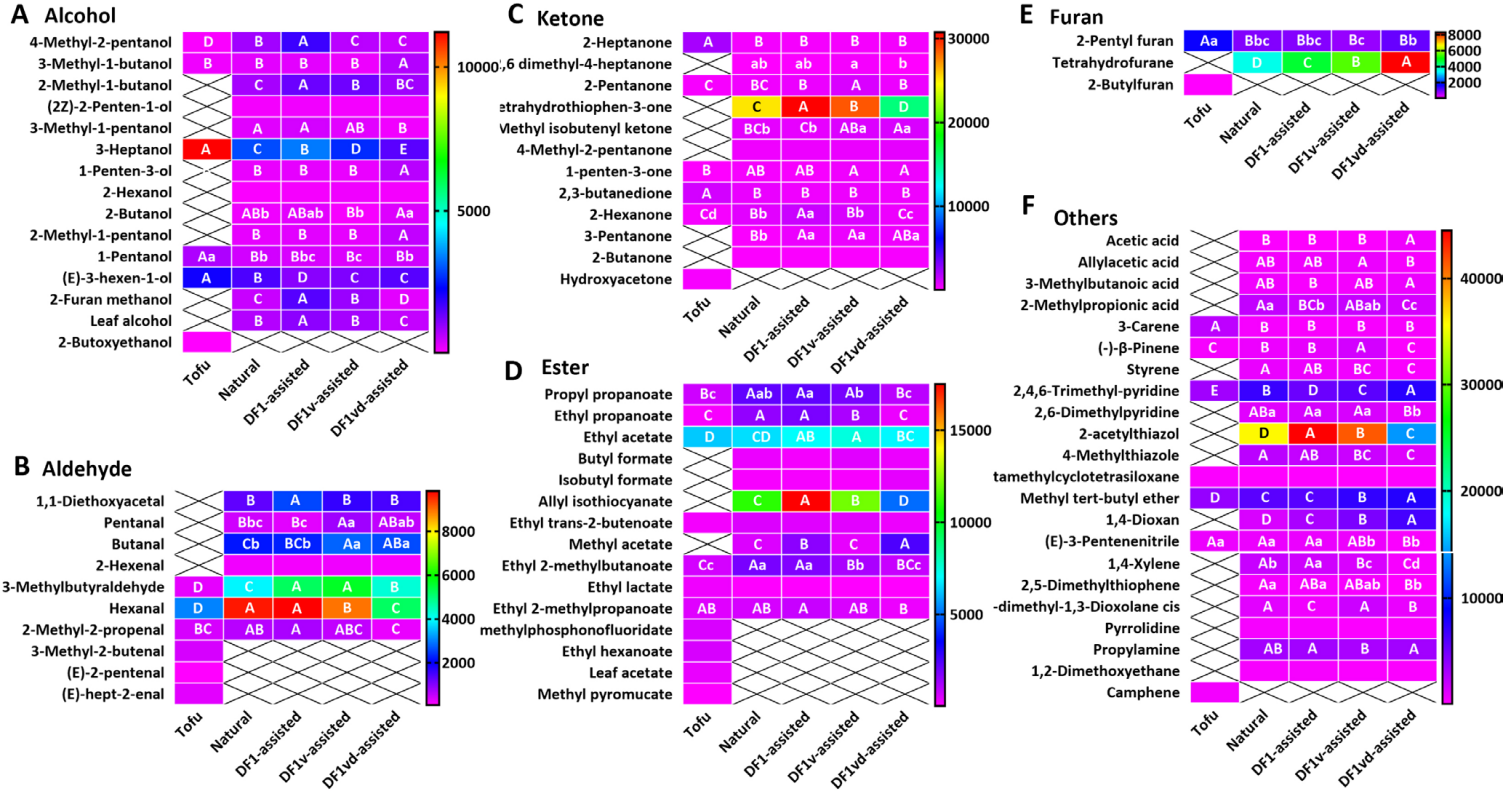
The types and contents of volatile flavor compounds significantly differed with assisted fermentation. (A) Types and contents of alcoholic flavor compounds in tofu or sufu products from the Natural, DF1‐assisted, DF1v‐assisted, and DF1vd‐assisted group products. (B) Types and contents of aldehyde flavor compounds in tofu or sufu products from the Natural, DF1‐assisted, DF1v‐assisted, and DF1vd‐assisted group products. (C) Types and contents of ketone flavor compounds in tofu or sufu products from Natural, DF1‐assisted, DF1v‐assisted, and DF1vd‐assisted group products. (D) Types and contents of ester flavor compounds in tofu or sufu products from the Natural, DF1‐assisted, DF1v‐assisted, and DF1vd‐assisted group products. (E) Types and contents of furan flavor compounds in tofu or sufu products from the Natural, DF1‐assisted, DF1v‐assisted, and DF1vd‐assisted group products. (F) Types and contents of other flavor compounds in tofu or sufu products from the Natural, DF1‐assisted, DF1v‐assisted, and DF1vd‐assisted group products. Different capital letters indicate highly significant differences (*p* < 0.01). Different lowercase letters indicate significant differences (*p* < 0.05). Natural fermentation: Natural fermentation; DF1‐assisted fermentation: DF1 strain‐assisted fermentation; DF1v‐assisted fermentation: DF1v strain assisted‐fermentation; DF1vd‐assisted fermentation: DF1vd strain‐assisted fermentation.

Alcoholic flavor substances can be divided into saturated and unsaturated substances, both of which are considered to impart flavor. As shown in Figure 5A, a total of 6 alcoholic flavor compounds, including 4‐methyl‐2‐pentanol, 3‐methyl‐1‐butanol, 3‐heptanol, 1‐penten‐3‐ol, 1‐pentanol and (E)‐3‐hexen‐1‐ol and 2‐butoxyethanol, were identified in tofu, among which 2‐butoxyethanol is a unique alcoholic flavor substance. Compared with tofu, 8 new alcoholic flavor substances, namely, 2‐methyl‐1‐butanol, (2Z)‐2‐penten‐1‐ol, 3‐methyl‐1‐pentanol, 2‐hexanol, 2‐butanol, 2‐methyl‐1‐propanol, 2‐furan methanol and leaf alcohol, formed in the sufu products. Overall, 3‐heptanol and (E)‐3‐hexen‐1‐ol were the most dominant alcoholic flavor compounds in the tofu and sufu products, but their contents significantly decreased throughout the fermentation process. The content of most alcoholic flavor compounds in the sufu products from the DF1vd‐assisted group was significantly lower than those from the Natural, DF1‐assisted, and DF1v‐assisted groups.

Aldehydes are generated mainly from amino acid degradation, the Strecker degradation reaction, and fatty acid oxidation (Zhang et al. 2023). As shown in Figure 5B, a total of 3 odor‐inducing aldehydes with carbon chain lengths mostly between C5 and C7 were identified in the tofu and sufu products, including 3‐methylbutyraldehyde, hexanal, and 2‐methyl‐2‐propenal. Fermentation resulted in a significant increase in the contents of the 3 smell‐inducing aldehydes relative to those in the tofu, but the contents were significantly lower in sufu products from the DF1vd‐assisted group than in those from the Natural, DF1‐assisted, and DF1v‐assisted groups. In addition, there were 3 other aldehyde flavor compounds in tofu, namely, 3‐methyl‐2‐butenal, (E)‐2‐pentenal, and (E)‐hept‐2‐enal. However, there were 4 different aldehyde flavor compounds in the sufu, namely, 1,1‐diethoxyacetal, pentanal, butanal, and 2‐hexenal. The aldehyde contents of sufu products produced by different bacteria were significantly different, and the different bacteria changed the content of aldehyde flavor compounds. Overall, hexanal was the most dominant aldehyde flavor compound in the tofu and sufu products.

Ketones are produced from fatty acid oxidation, amino acid degradation, and the Maillard reaction and have higher thresholds than aldehydes (Fu et al. 2022). As shown in Figure 5C, a total of 5 ketone flavor compounds, including 2‐heptanone, 2‐pentanone, 1‐penten‐3‐one, 2,3‐butanedione, and 2‐hexanone, were identified in both the tofu and sufu products. Hydroxyacetone is a unique ketone flavor compound in tofu. Compared with those in tofu, 6 new ketone flavor compounds were formed in the sufu products, namely, 2,6‐dimethyl‐4‐heptanone, tetrahydrothiophen‐3‐one, methyl isobutenyl ketone, 4‐methyl‐2‐pentanone, 3‐pentanone, and 2‐butanone. Overall, 2‐heptanone and 2,3‐butanedione were the most dominant ketone flavor compounds in tofu. Tetrahydrothiophen‐3‐one was the most dominant ketone flavor compound in the sufu products, and its content was significantly lower in the sufu products from the DF1vd‐assisted group than in those from the Natural, DF1‐assisted, and DF1v‐assisted groups.

Esters have a pleasant fruit flavor and are generally formed from the esterification of carboxylic acids and alcohols. As shown in Figure 5D, a total of 7 ester flavor compounds, including propyl propanoate, ethyl propanoate, ethyl acetate, ethyl trans‐2‐butenoate, ethyl isobutyrate, ethyl lactate, and ethyl 2‐methylpropanoate, were identified in the tofu and sufu products. Of these, ethyl acetate imparts a pleasant fruity ethereal aroma, which provides a pleasant aroma to the sufu products. In tofu, there are 4 other ester flavor compounds, namely, isopropyl methylphosphonofluoridate, ethyl hexanoate, leaf acetate, and methyl pyromucate. In the sufu products, there were 4 other ester flavor compounds, namely, butyl formate, isobutyl formate, allyl isothiocyanate, and methyl acetate. These results indicated that microbial fermentation resulted in the formation of new ester flavor compounds, such as butyl formate, isobutyl formate, allyl isothiocyanate, and methyl acetate. However, at the same time, isopropyl methylphosphonofluoridate, ethyl hexanoate, leaf acetate, and methyl pyromucate disappeared. Ethyl acetate, propyl propanoate, ethyl isobutyrate, and ethyl hexanoate were important ester flavor compounds in the tofu. Ethyl acetate and allyl isothiocyanate were important ester flavor compounds in the sufu products.

Furans impart a caramelly, sweet, and baked flavor. 2‐Pentyl furan, a type of noncarbonyl oxidation product derived from linoleic acid and other n‐6 PUFAs, also imparts a pleasant flavor to meat products and was detected in both tofu and sufu products, as shown in Figure 5E. Through fermentation, a new furan, tetrahydrofurane, was produced, and it became the most dominant furan flavor compound, which was significantly more abundant in the sufu products from the DF1vd‐assisted group than in those from the Natural, DF1‐assisted, and DF1v‐assisted groups.

Moreover, there were 9 other flavor compounds in tofu and 21 other flavor compounds in the sufu products (Figure 5F). Overall, the types and contents of these flavor compounds were greater in the sufu products than in tofu. Compared with those in the other groups, the contents of six flavor compounds, namely, acetic acid, 3‐methylbutanoic acid, 2,4,6‐trimethyl‐pyridine, methyl tert‐butyl ether, 1,4‐xylene, and propylamine, were greater in the sufu products from the DF1vd‐assisted group.

All VOCs with ROAVs > 1 are shown in Table 3. There were 16 of these compounds in the sufu products from the DF1vd‐assisted group, 15 in those from the DF1v‐assisted group, 12 in those from the DF1‐assisted group, 12 in those from the Natural group, and 11 in the tofu. The characteristic VOCs included pentanal, 3‐methylbutyraldehyde, hexanal, 2‐methyl‐2‐propenal, 1‐penten‐3‐one, 2,3‐butanedione, ethyl propanoate, ethyl acetate, allyl isothiocyanate, ethyl isobutyrate, ethyl 2‐methylbutanoate, 2‐pentyl furan, 2‐acetylthiazol, 2,4,6‐trimethylpyridine, 2,6‐dimethylpyridine, and methyl tert‐butyl ether. Furthermore, the ingredients that contributed the most to flavor were ethyl isobutyrate and ethyl 2‐methylbutanoate. Ethyl 2‐methylpropanoate was the most dominant ester flavor compound in the tofu, and ethyl isobutyrate was the most dominant ester flavor compound in the sufu products. (E)‐hept‐2‐enal was the most dominant aldehyde flavor compound in the tofu or sufu products from the Natural and DF1 groups, but 3‐methylbutyraldehyde was the most dominant aldehyde flavor compound in the DF1v sufu and DF1vd sufu groups. 2,3‐Butanedione was the most dominant ketone flavor compound in tofu, but 1‐penten‐3‐one was the most dominant flavor compound in the sufu products. Alcoholic flavor substances had a weak contribution to the flavor of tofu and sufu products.

### 3.7 The Content of Nonbeneficial BAs in Sufu Products–Assisted Fermentation Was Not Considerably Increased

**FIGURE 6:**
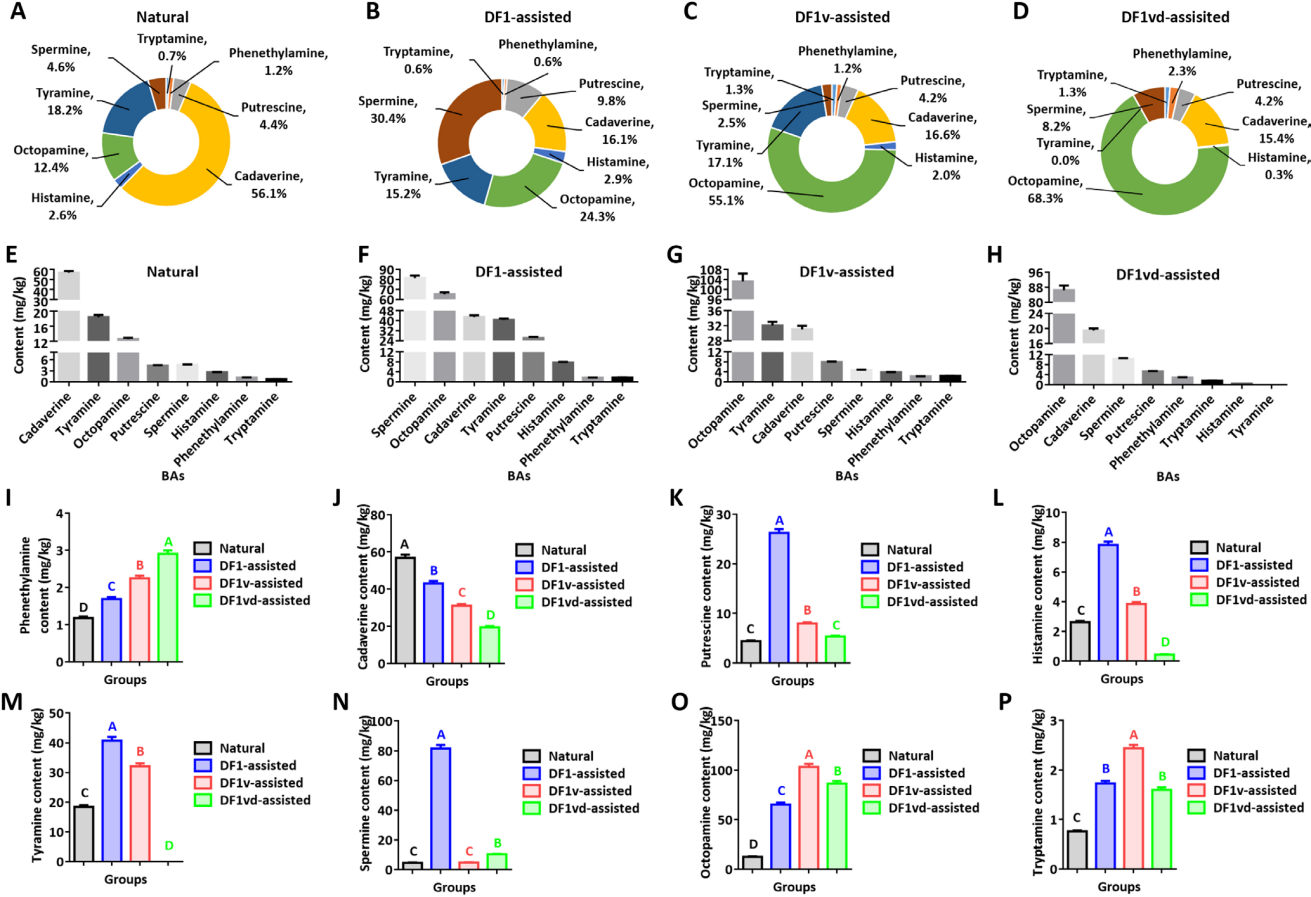
The content of nonbeneficial BAs did not increase in sufu products produced via assisted fermentation. (A) Proportion of BAs in the sufu products from the Natural group. (B) Proportion of BAs in the sufu products from the DF1‐assisted fermentation group. (C) Proportion of BAs in the sufu products from the DF1v‐assisted group. (D) Proportion of BAs in the sufu products from the DF1vd‐assisted group. (E) The contents of BAs in the sufu products from the Natural group. (F) The contents of BAs in the sufu products from the DF1‐assisted group. (G) The contents of BAs in the sufu products from the DF1v‐assisted group. (H) The contents of BAs in the sufu products from the DF1vd‐assisted group. (I‐P) The contents of 8 BAs, namely, phenethylamine (I), cadaverine (J), putrescine (K), histamine (L), tyramine (M), spermine (N), octopamine (O), and tryptamine (P), were significantly different in the sufu products from the Natural, DF1‐assisted, DF1v‐assisted, and DF1vd‐assisted groups. Different capital letters indicate highly significant differences (*p* < 0.01). Different lowercase letters indicate significant differences (*p* < 0.05). Natural fermentation: Natural fermentation; DF1‐assisted fermentation: DF1 strain‐assisted fermentation; DF1v‐assisted fermentation: DF1v strain‐assisted fermentation; DF1vd‐assisted fermentation: DF1vd strain‐assisted fermentation.

Eight BAs were detected in the samples, and the results are shown in Figure 6. Five BAs, including cadaverine, putrescine, spermine, octopamine, and tyramine, were relatively abundant. Both the BA composition and content in the sufu products from the Natural, DF1‐assisted, DF1v‐assisted, and DF1vd‐assisted groups were significantly different. The total BA contents (wet weight basis) of the sufu products from the Natural, DF1‐assisted, DF1v‐assisted, and DF1vd‐assisted groups were 101.49 ± 3.0 mg/kg, 268.11 ± 8.0 mg/kg, 187.76 ± 5.6 mg/kg, and 126.59 ± 3.80 mg/kg, respectively. There was less BA produced in the sufu products from the DF1vd‐assisted group than in those from the DF1‐assisted and DF1v‐assisted groups but more than in those from the Natural group (Figure 6A–H). Among the sufu products from the Natural group, cadaverine accounted for the greatest proportion and content of total BAs (56.1%, 56.90 ± 1.71 mg/kg), followed by tyramine, octopamine, spermine, putrescine, histamine, phenethylamine, and tryptamine (Figure 6A,E). However, in the sufu products from the DF1‐assisted, DF1v‐assisted, and DF1vd‐assisted groups, the BA with greatest proportion and content was spermine (30.4%, 81.55 ± 22.45 mg/kg) (Figure 6B,F), octopamine (55.1%, 103.3 ± 3.10 mg/kg) (Figure 6C,G), and octopamine (68.3%, 886.46 ± 2.59 mg/kg) (Figure 6D,H), respectively. The content of phenethylamine decreased in the order of Natural < DF1‐assisted < DF1v‐assisted < DF1vd‐assisted, and significant differences were detected among these groups (Figure 6I). The content of cadaverine showed the opposite trend (Natural > DF1‐assisted > DF1v‐assisted > DF1vd‐assisted) and significantly differed among these groups (Figure 6J). The contents of putrescine, histamine, and tyramine tended to decrease in the order DF1‐assisted > DF1v‐assisted>Natural > DF1vd‐assisted and were significantly different among these groups (Figure 6K–M). The content of spermine tended to decrease in the order of DF1‐assisted > DF1vd‐assisted > DF1v‐assisted ≈ Natural, and significant differences were detected among these groups (Figure 6N). The octopamine content tended to decrease in the order of DF1v‐assisted > DF1vd‐assisted > DF1‐assisted>Natural and significantly differed among these groups (Figure 6O). The content of tryptamine tended to decrease in the order DF1v‐assisted > DF1‐assisted ≈ DF1vd‐assisted>Natural and significantly differed among these groups (Figure 6P).

## 4 Discussion

Many unidentified microorganisms are present in traditional sufu, including different bacteria and fungi (Luo et al. 2023; Zhao et al. 2023). Some microorganisms change the composition and content of nutrients by fermenting tofu and producing secondary metabolites that are beneficial to human health (Do Prado et al. 2022). However, some microorganisms may consume probiotic factors and produce hazardous factors, which are harmful to consumers (Liu, Chen et al. 2022). In this study, we screened and isolated safe bacteria with the ability to produce protease from traditional sufu. The three strains DF1, DF2, and DF3 are probiotics, and DF4 can pose risks to human health according to its genetic makeup and related application research. The DF1 strain with dominant protease activity was chosen for the following study.

In most cases, fungi, especially *Mucor*, are the dominant microorganisms involved in the production process of traditional sufu because of their unique mycelial structure and ability to secrete protease (Wan et al. 2020; Xie et al. 2023). However, the fermentation process to prepare traditional sufu from tofu to pehtzes takes more than 15 days, followed by a longer maturation period of 3–6 months (Wu et al. 2022). The long production period undoubtedly hinders the industrialization of sufu production, increases the time cost of sufu production, and even increases the concentration of harmful microorganisms. In this study, the fermentation period of sufu pehtzes was significantly shortened to 6–8 days through assisted fermentation using the domesticated strain DF1vd, with the contents of water‐soluble protein and amino acid nitrogen reaching a plateau, indicating thorough fermentation. Moreover, the maturation period of the sufu product was significantly shortened to 20 days, which improved the quality of the sufu product. The sufu product also has a full aroma, with a strong umami, sour, and beany smell. However, the moldy smell was weaker. This kind of sufu product obtained through DF1vd‐assisted fermentation may become favored by the wider public, increasing its market prospects.

In the process of tofu fermentation and sufu maturation, the crude components of tofu are degraded into small molecules, such as amino acids, fatty acids, and oligosaccharides, under the action of microorganisms and their secreted enzymes (Moy, Lu, and Chou 2012; Xie et al. 2023). The degradation of macromolecules leads to a weakening of the texture profile and an increase in total acid content (He, Chen, and Chung 2022; Li et al. 2021). In this study, although the texture profiles of the products were relatively weak, the sufu product from DF1vd‐assisted fermentation could maintain its shape. Because amino acids were produced and released, the total acid content increased, corresponding to increased sour and umami aromas, which improved the palatability of the product to consumers and gave the product a good mouth‐feel.

The HAAs that accumulated in the sufu partly originated from the hydrolysis of soy protein, and the other part was derived from microbial fermentation. Microbial fermentation increased the vitality and growth of microbes, which produced large amounts of proteases, lipases, and amylases that can hydrolyze the proteins in soybeans into amino acids through a series of biochemical processes. Monosodium glutamate (MSG)‐like amino acids (Glu. and Asp.) are known to be the key compounds responsible for the umami flavor. Interactions between sweet amino acids (represented by Thr., Ser., Pro., Gly., and Ala.) and IMP (inosine monophosphate) could be another pathway through which umami flavors are enhanced (Imada et al. 2014), with such amino acids being present in relatively high amounts in assisted fermentation products. Moreover, several bitter amino acids were also detected in the sufu products. The content of bitter amino acids accounted for approximately 47.51%–48.49% of the total amino acids. Interestingly, a previous study confirmed that bitter amino acids could actually enhance the umami flavor. Microbial fermentation by the DF1vd strain was particularly necessary for the production of sufu with a unique taste. Our results are in agreement with those of a previous report (Yao, Xu, Wu, Wang, Zhu et al. 2021).

It has been shown that the number of flavor compounds increases when tofu is processed into sufu (Liang et al. 2019). This might occur because the endogenous enzymes in tofu and the enzymes secreted by microorganisms, such as proteases, glutaminases, peptidases, lipases, cellulases, hemicellulases, α‐amylases, and β‐glucosidases, have an activating effect on sufu during fermentation (Chen et al. 2023; Wei et al. 2023). These enzymes break down the amino acids, proteins, lipids, and carbohydrates present in the raw materials into short peptides, free amino acids, fatty acids, and sugars to form the unique flavor or flavor precursor compounds of sufu (Liu, Chen et al. 2022).

The main volatile compounds of sufu were esters, alcohols, and aldehydes, which is in accordance with previous research (Chen et al. 2022). Recent studies have shown that ethyl propanoate, ethyl isobutyrate, and ethyl 2‐methylbutanoate are the key characteristic flavor compounds of sufu (Chen et al. 2023; Xie et al. 2018). Ethyl propanoate is associated with sweet, pineapple‐like, floral, and green‐leafy flavors (Wang et al. 2022). Methyl 2‐methylbutanoate imparts sweet, fruity, blueberry‐like, floral, and caramelly aromas (Forney et al. 2022). 2‐heptanone, produced by linoleic acid oxidation, imparts a cinnamon‐like and fruity flavor (Yang et al. 2021), whereas 1‐penten‐3‐one imparts a unique fungal flavor. These flavor compounds jointly contribute to the unique sweet, floral, fruity, pungent, caramelly, and malty flavor profiles of sufu.

The DF1vd sufu product was remarkably rich in flavor compounds with ROAV > 1, which meant that DF1vd was an important bacterium for improving sufu flavor. With the exception of histamine, government regulatory thresholds for the maximum allowable levels of biogenic amines in food have not been set. The upper allowable limits of histamine in foods for human consumption have been suggested by the U.S. Food and Drug Administration, the European Food Safety Authority, and the Chinese government (GB 2733–2015) to be 50 mg/kg, 50–200 mg/kg, and 200–400 mg/kg, respectively. In this study, the BA contents in the sufu products were different among the four groups, all of which were below the maximum contaminant level (MCL; 900 mg/kg), which was consistent with previous recommendations (Silla Santos 1996). The histamine concentrations within the four products were lower than the above three allowable limits, and the concentration of histamine in the DF1vd group was approximately 6.08 times lower than that in the Natural group. The concentrations of cadaverine, octopamine, and spermine in the DF1vd‐assisted group were approximately 2.92, 6.89, and 2.24 times lower than those in the Natural group, respectively. These results suggest that DF1vd‐assisted fermentation may inhibit the production of nonbeneficial BAs in sufu products.

Octopamine is considered to prevent obesity and type 2 diabetes (Brial et al. 2019; Lee et al. 2022). The various physiological effects of spermine on the regulation of animal reproduction, endocrine function, digestion, and absorption have been elucidated (Hu et al. 2021). In this study, we found that the concentrations of octopamine and spermine in the sufu products from the DF1vd‐assisted group were approximately 6.89 and 2.24 times greater than those from the Natural group, respectively. These results suggest that DF1vd‐assisted fermentation may promote the production of BAs beneficial for humans in sufu products.

## 5 Conclusions

In summary, a *Bacillus subtilis* strain (DF1) and derivative strains (DF1v and DF1vd) with protease production capacity and industrial application potential were obtained. When these strains were applied in assisted fermentation, the quality and flavor of sufu products were improved due to the thorough degradation of soy protein, stable texture, an abundance of crude components, an increase in HHAs content, refinement of the flavor composition for good taste, and an increase in the production of beneficial BAs such as octopamine and spermidine rather than nonbeneficial BAs, at a shorter fermentation and postripening time. The intake of improved sufu products is conducive to human health.

## Author Contributions

**Junfei Xu:** funding acquisition (equal), investigation (equal), methodology (equal), writing – original draft (equal). **Aixiang Hou:** investigation (equal), methodology (equal), writing – review and editing (equal). **Wenqi Li:** investigation (equal), software (equal). **Binbin Chen:** investigation (equal), methodology (equal). **Hong Wu:** investigation (equal), methodology (equal). **Huan Tan:** investigation (equal), methodology (equal). **Zhihong Xiao:** investigation (equal), methodology (equal), software (equal). **Xianjin Wu:** funding acquisition (equal), investigation (equal), methodology (equal). **Juzuo Zhang:** conceptualization (equal), project administration (equal), supervision (equal), writing – review and editing (equal).

## Ethics Statement

The human sensory study was approved by the Science and Technology Ethical Committee of Huaihua University (2021EK04104). Sensory evaluation was performed strictly according to the ethical and professional practices in the IFST guidelines, ISO 8586‐2012 and SB/T 10170–2007.

## Consent

The authors confirm thatall participants provided informed consent and that their rights and privacy were protected during the execution of the research, e.g., study requirements and risks were fully disclosed, no participants were coerced into participating, written or verbal consent was obtained from all participants, and the participants could withdraw from the study at any time.

## Conflicts of Interest

The authors declare no conflicts of interest.

## Data Availability

The data that support the findings of this study are available upon request from the corresponding authors.
